# Multi-threshold image segmentation for melanoma based on Kapur’s entropy using enhanced ant colony optimization

**DOI:** 10.3389/fninf.2022.1041799

**Published:** 2022-11-01

**Authors:** Xiao Yang, Xiaojia Ye, Dong Zhao, Ali Asghar Heidari, Zhangze Xu, Huiling Chen, Yangyang Li

**Affiliations:** ^1^School of Computer Science and Technology, Changchun University of Science and Technology, Changchun, China; ^2^School of Statistics and Mathematics, Shanghai Lixin University of Accounting and Finance, Shanghai, China; ^3^College of Computer Science and Technology, Changchun Normal University, Changchun, China; ^4^School of Surveying and Geospatial Engineering, College of Engineering, University of Tehran, Tehran, Iran; ^5^College of Computer Science and Artificial Intelligence, Wenzhou University, Wenzhou, China; ^6^Department of Pathology, The First Affiliated Hospital of Wenzhou Medical University, Wenzhou, China

**Keywords:** melanoma, multi-threshold image segmentation, Kapur’s entropy, swarm intelligence, ant colony algorithm

## Abstract

Melanoma is a malignant tumor formed by the cancerous transformation of melanocytes, and its medical images contain much information. However, the percentage of the critical information in the image is small, and the noise is non-uniformly distributed. We propose a new multi-threshold image segmentation model based on the two-dimensional histogram approach to the above problem. We present an enhanced ant colony optimization for continuous domains (EACOR) in the proposed model based on the soft besiege and chase strategies. Further, EACOR is combined with two-dimensional Kapur’s entropy to search for the optimal thresholds. An experiment on the IEEE CEC2014 benchmark function was conducted to measure the reliable global search capability of the EACOR algorithm in the proposed model. Moreover, we have also conducted several sets of experiments to test the validity of the image segmentation model proposed in this paper. The experimental results show that the segmented images from the proposed model outperform the comparison method in several evaluation metrics. Ultimately, the model proposed in this paper can provide high-quality samples for subsequent analysis of melanoma pathology images.

## Introduction

Melanoma is a malignant tumor arising from the malignant transformation of melanocytes. While melanoma is less common than other skin cancers, it is responsible for nearly 10,000 deaths in the United States each year alone (Albittar et al., 2020). Due to the similarity of cutaneous melanoma to benign nevi can easily be overlooked and misdiagnosed in the pathological diagnosis. Accurate pathological diagnosis plays an important role in the therapy of melanoma (Ahn et al., 2017; Bi et al., 2017; Al-Masni et al., 2018). To improve the diagnostic accuracy, the concept of computer-assisted pathological diagnosis of initial malignant melanoma has been intensively investigated in recent years. Lee et al. (2018) proposed a computer-aided diagnostic process that effectively differentiated melanoma images from non-melanoma images through feature extraction, selection, and classification. Song et al. (2020) designed an end-to-end multitasking deep learning framework for automated analysis of melanoma diagnosis. Chen W. et al. (2020) used logistic regression and the Newton-Raphson method to effectively discriminate between melanoma and benign nevus. In general, computer-aided diagnosis can be divided into image acquisition (Lafci et al., 2022), pre-processing (Ilesanmi et al., 2021), image segmentation (Wang S. et al., 2022), feature extraction (You et al., 2022) and classification (Hu et al., 2021; Yu et al., 2021). The image segmentation technique is key to further analysis of melanoma pathology images, which can provide quality image samples for subsequent image analysis and ultimately improve the accuracy of melanoma diagnosis (Pennisi et al., 2016; Kassem et al., 2021).

In order to be able to provide quality material for subsequent image processing, we employed an image segmentation method with the advanced threshold optimization technique. In this study, an enhanced ant colony algorithm (EACOR) based on the soft besiege strategy and the chase strategy is proposed to obtain a more reasonable threshold segmentation scheme. Furthermore, to obtain a more reasonable solution that retains the maximum amount of information between the target and the background, Kapur’s entropy is used as the objective function for EACOR to evaluate the threshold sets. In addition, the non-local mean two-dimensional histogram method is used to exploit the spatial information of the image to reduce noise interference. The threshold optimization ability and segmentation ability of the proposed model are verified in this paper. A series of global optimization experiments on the IEEE CEC2014 benchmark test function (Liang et al., 2013) is used to validate whether EACOR is a suitable threshold search method. To test the image segmentation model, we set up segmentation experiments on the melanoma dataset at five threshold levels of 4, 8, 12, 16, and 20. Feature similarity index (FSIM) (Zhang et al., 2011), structural similarity index method (SSIM) (Wang et al., 2014), and peak signal to noise ratio (PSNR) (Huynh-Thu and Ghanbari, 2008) is used to evaluate the segmentation experiments to feedback on a more comprehensive and objective result. The experimental results show that the model proposed in this paper can perform the task of image segmentation excellently. It provides high-quality samples for subsequent melanoma pathology image segmentation. In summary, the contributions of this paper are as follows:

♦ A novel ant colony algorithm with the soft besiege and chase strategies is proposed. Its powerful optimization capability is applied to the segmentation of melanoma pathology images, and segmentation results of high quality are obtained.
♦ The performance of EACOR is compared with some excellent peers and variants.
♦ EACOR improves the original ant colony algorithm’s convergence speed, accuracy, and ability to jump out of the local optimum on complex functions.
♦ The quality of the segmented images is evaluated comprehensively, and the proposed threshold optimization method can improve the efficiency of threshold search.

The remainder of the paper is organized as follows: related works are discussed in Section “Related works.” Section “Overview of relevant methods” reviews the multi-threshold segmentation method and the original ant colony algorithm. Section “EACOR-based segmentation model” introduces the EACOR-based multilevel threshold segmentation method. Section “Experiments and results analysis” shows and analyzes the experimental results of the benchmark function and image segmentation. The model proposed in this paper is discussed in Section “Discussions.” In Section “Conclusion and future works,” the work of the paper and the next research plan are summarized.

## Related works

### Threshold segmentation method

As one of the most common image segmentation methods, threshold segmentation usually determines the gray scale range of the segmented image based on pixel gray scale or other ordered metrics. The segmentation is then completed based on a histogram of the grayscale, mean grayscale, non-local mean, etc. According to the objective function, threshold segmentation can be divided into minimum error, maximum between-class variance (Otsu), maximum entropy, etc. The most widely used of these is the maximum entropy method and Otsu. Threshold segmentation does not rely on *a priori* knowledge of the image and has low computational complexity. In addition, threshold segmentation is suitable for processing some images where the gray scale reflects the target of interest. Therefore, this method is often applied to the problem of processing medical images. However, the increase in threshold level according to the medical diagnosis requirements make the objective function complex and increases computational cost. Recently, some researchers have found that taking advantage of swarm intelligence algorithms can reduce computational consumption. As a result, the multi-level threshold image segmentation method with swarm intelligence optimization has attracted more attention.

For example, Siri et al. (2020) proposed a novel multi-threshold liver segmentation model based on the ‘Slope Difference Distribution’ of image histogram, and the segmentation of the CT images of the liver is satisfactory. The Jaccard Coefficient results with the popular method demonstrated that this model could be more accurate for lung CT images. Li W. et al. (2020) presented a multi-threshold segmentation-based genetic algorithm for fine segmentation of medical images. The experimental results show that the feature extraction model based on the segmentation method had a higher region recognition rate. Renugambal and Selva Bhuvaneswari (2021) used Kapur’s entropy and a modified moth flame optimization algorithm to differentiate between gray matter, white matter, and cerebrospinal fluid. The objective function values, the PSNR, and the computational cost were all satisfactory. Patra et al. (2021) used a multilayer threshold segmentation method based on a student psychology-based optimizer for lesion detection on breast dynamic contrast-enhanced magnetic resonance imaging. The accuracy of the segmented image after feature extraction was 99.44%. Chen et al. (2022) designed an enhanced shuffled frog leaping algorithm for multi-threshold image segmentation of breast cancer. The method was used for multi-threshold image segmentation of breast invasive ductal carcinoma, and the images obtained by the method performed well in terms of FSIM, PSNR, and SSIM. As a result, swarm intelligence optimization has been widely used for solving multi-threshold image segmentation problems.

Analysis of previous literature shows that the multi-threshold image segmentation method based on the optimization algorithm is an image segmentation method with excellent segmentation performance, which is suitable for segmenting medical images. Furthermore, such segmentation methods still have great potential due to the performance of threshold optimization methods.

### Optimization method

The swarm intelligence algorithm is a flexible, gradient-independent method. Solving for the optimal threshold reduces the risk of obtaining sub-optimal or inferior solutions. The field of optimization of swarm intelligence algorithms is not restricted to a specific problem, and there are no special requirements for the objective function. Based on these advantages, it has become one of the most popular optimization methods. Common swarm intelligence include: differential evolution algorithm (DE) (Storn and Price, 1997), stochastic fractal search (SFS) (Salimi, 2015), ant colony algorithm for continuous domain problems (ACOR) (Socha and Dorigo, 2008), grey wolf optimizer (GWO) (Mirjalili et al., 2014), moth-flame optimization (MFO) (Mirjalili, 2015), whale optimization algorithm (WOA) (Mirjalili and Lewis, 2016), Runge Kutta optimizer (RUN) (Ahmadianfar et al., 2021), tree-seed algorithm (TSA) (Kiran, 2015), firefly algorithm (FA) (Yang, 2010), hunger games search (HGS) (Yang et al., 2021), Harris hawks optimization (HHO) (Heidari et al., 2019b), sine cosine algorithm (SCA) (Mirjalili, 2016), slime mould algorithm (SMA) (Li S. et al., 2020), colony predation algorithm (CPA) (Tu et al., 2021), weighted mean of vectors (INFO) (Ahmadianfar et al., 2022), and cuckoo search algorithm (CS) (Yang and Deb, 2010). The above methods have been applied to a particular area with excellent performance. However, the increase in dimensionality and complexity leads to low convergence accuracy and a higher probability of obtaining a locally optimal solution. Therefore, improving existing algorithms is one of the most common ways to enhance their optimization performance.

For example, a boosted bat algorithm (CDLOBA) (Yong et al., 2018), the chaotic bat algorithm (CBA) (Adarsh et al., 2016), the hybridizing grey wolf optimization with differential evolution (HGWO) (Zhu et al., 2015), the hybrid algorithm (ASCA-PSO) that combines SCA with PSO (Issa et al., 2018), the hybridizing sine cosine algorithm with differential evolution (SCADE) (Nenavath and Jatoth, 2018), the modified SCA based on neighborhood search and greedy Lévy mutation (m_SCA) (Qu et al., 2018), the improved GWO algorithm (IGWO) exploits the powerful exploratory power of the hierarchical mechanism (Cai et al., 2019), the efficient boosted grey wolf optimizers (OBLGWO) (Heidari et al., 2019a), the A-C parametric whale optimization Algorithm (ACWOA) (Khashan et al., 2018), the mutative whale-inspired optimization methods with multi-strategy (BMWOA) (Luo et al., 2019), the multi-population and DE assisted Harris hawks optimization (CMDHHO) (Chen H. et al., 2020). Therefore, based on an analysis of previous literature, we believe that focused improvement of the original algorithm is a viable option. These methods have excellent performance in dealing with some optimization problems, such as economic emission dispatch problem (Dong et al., 2021), image segmentation (Hussien et al., 2022; Yu et al., 2022b), feature selection (Hu J. et al., 2022; Liu et al., 2022), robust optimization (He et al., 2019, 2020), scheduling problems (Gao et al., 2020; Han et al., 2021; Wang G. G. et al., 2022), multi-objective problem (Hua et al., 2021; Deng et al., 2022d), plant disease recognition (Yu et al., 2022a), complex optimization problem (Deng et al., 2022b), train scheduling (Song et al., 2023), resource allocation (Deng et al., 2022a), airport taxiway planning (Deng et al., 2022c), optimization of machine learning model (ling Chen et al., 2014), medical diagnosis (Chen et al., 2016; Wang et al., 2017), and solar cell parameter identification (Ye et al., 2021). But there are still some issues to be considered in these methods. For example, Niu et al. (2019) found after repeated experiments that GWO was uncertain when dealing with optimization problems. The optimization capability of GWO is significantly better than other methods only when the optimal solution of the optimization problem is zero. When the optimal value is far from zero, the performance of GWO gradually decreased. It means that the algorithm performance is unstable for real-world applications. Furthermore, a series of studies based on algorithm structure and core ideas question the innovativeness of some popular algorithms including intelligent water drops algorithm, firefly algorithm, black holes optimization algorithm, whale optimization algorithm and sine cosine algorithm. Some of these studies have argued that these methods are simply a novel modification of previous methods, confusing the researcher’s understanding of the nature of the algorithm (Piotrowski et al., 2014; Camacho-Villalón et al., 2018; Camacho Villalón et al., 2020).

Based on the above factors, the originality of the ant colony optimization algorithm (ACO) is supported by a scientifically complete theory. The traditional ACO is mainly applied to solve discrete problems. Subsequently, Socha and Dorigo (2008) proposed ACOR for continuous domains, known as ACOR. ACOR and its variants are widely used in various fields. ACOR is a classical swarm intelligence algorithm with a simple structure and high robustness. ACOR and its variants are widely used in various fields. Juang and Chang (2011) applied the improved ACOR algorithm (ECACOR) to the field of dynamic plant control. Zhang et al. (2013) proposed applying the homogenous continuous ant colony optimization algorithm (HACOR) to the one-dimensional coupled radiation and thermal conductivity heat transfer inverse problem. Gao (2014) solved the landslide forecasting problem by combining the artificial immune system with ACOR. Jero et al. (2016) applied ACOR to Electrocardiography steganography. Ma et al. (2019) proposed an adaptive hybrid ACO for the classification problem. Omran and Al-Sharhan (2019) applied two variants of his proposed ACOR to practical engineering optimization problems.

Based on an analysis of previous literature, ACOR can obtain better result when dealing with different optimization tasks. However, ACOR still has potential for improvement. When solving high-dimensional problems or complex multi-peaked problems, the convergence accuracy of ACOR may appear insufficient, and the probability of obtaining a locally optimal solution increase. If ACOR is used as a thresholding search method for segmentation models, it may reduce the effectiveness of image segmentation. Based on the above literature analysis, it can be concluded that a multi-threshold segmentation method with excellent performance is still necessary. Therefore, this study proposes an enhanced ACOR method (EACOR) based on the soft besiege and chase strategies.

## Overview of relevant methods

### Multi-threshold image segmentation

#### Two-dimensional histogram

The non-local means (NLM) two-dimensional histogram (Buades et al., 2005) is an essential method for image denoising. The basic principle of NLM filtering is like that of means filtering. They both achieve denoising by averaging the pixels around the current pixel point, except that a weighting strategy is added to the NLM filtering. NML method can achieve denoising and preserve the details of the image edges. The value of NLM *O*(*p*) can be obtained according to Eq. 1.


(1) $$O\left(p\right)=\frac{\sum_{q\in I}X\left(q\right)\omega \left(p,q\right)}{\sum_{q\in I}\omega \left(p,q\right)}$$

where *p* and *q* are two pixels in image *I*, respectively. *X*(*p*) and *X*(*q*) represent the values of *p* and *q*, respectively. The Gaussian weighting function ω(*p, q*) is defined as shown in Eq. 2.


(2) $$\omega \left(p,q\right)=exp^{-\frac{{\left|\mu \left(q\right)-\mu \left(p\right)\right|}^{2}}{\sigma^{2}}}$$

where σ is the standard deviation of the Gaussian weighting function. μ(*p*), μ(*q*) denote the local means of *p, q* pixels, respectively, obtained from Eq. 3.


(3) $$\mu \left(x\right)=\frac{1}{n\times n}\sum_{i\in S\left(x\right)}I\left(i\right)$$

where *x* is a pixel in the image *I, S*(*x*) is a square filter of size *n* × *n* around pixel *x*.

Suppose an image *I*(*x*, *y*) with gray level *L* and size *M* × *N, x* ∈ [1, *M*], *y*∈ [1, *N*]. For each pixel in *I*, the corresponding grayscale value *f*(*x*, *y*) and NLM *g*(*x*, *y*) can be calculated. Therefore, each pixel in the original image *I*(*x*, *y*) will be associated with two dimensions: grayscale and NLM. Moreover, we can also obtain the number of *h*(*i*, *j*) pixels with the same grayscale and NLM in (*s*, *t*), which is calculated by Eq. 4.


(4) $$h\left(i,j\right)=c_{i,j}$$

where *i, j* denote the grayscale value *f*(*x*, *y*) and the nonlocal mean *g*(*x*, *y*) of (*x*, *y*) pixels. Both *i* and *j* are in the range of 0 to *L*1. *c*_*i,j*_ is the number of pixels with grayscale value and NLM of (*i*, *j*). Two-dimensional histogram of the image is obtained by normalizing Eq. (5) according to *h*(*i*, *j*).


(5) $$P_{i,j}=\frac{h\left(i,j\right)}{M\times N},i,j=0,1,2,\ldots ,L-1,\sum_{i=0}^{L-1}\sum_{j=0}^{L-1}P_{i,j}=1$$

**FIGURE 1:**
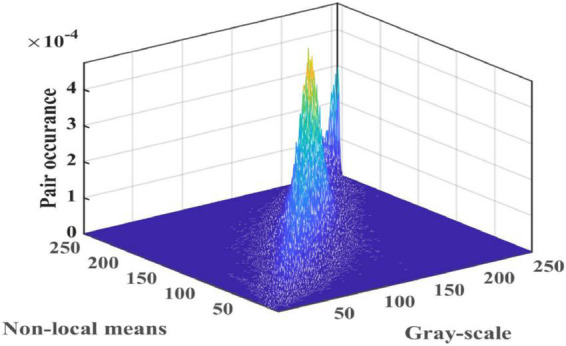
Three-dimensional view of a 2D histogram.

The grayscale and NLM values are used as the horizontal and longitude axes of the two-dimensional histogram. The number of normalized pixels is used as the vertical axis of the two-dimensional histogram, as shown in Figure 1.

#### Kapur’s entropy

Entropy is a physical quantity that measures a certain distribution, and a higher entropy value means that the distribution is more uniform. In order to fully consider the source independence and be able to well extract the feature signal in the picture, Kapur’s entropy is selected to measure the amount of information in the target region and background region. The larger Kapur’s entropy indicates the higher quality of image segmentation. The method of MTIS using Kapur’s entropy can be described as follows: {*t*_1_, *t*_2_ …, *t*_*T*_} represents the grayscale values of the grayscale image and {*s*_1_, *s*_2_ …, *s*_*T*_} characterizes the grayscale values of the non-local mean image. The objective function is expressed as calculating the entropy of *T* + 1 image segmentations and then summing them. The expression for the objective function *F* of Kapur’s entropy is shown in Eqs. 6, 7.


(6) $$F\left(th_{1},th_{2},\ldots ,th_{T}\right)=H_{0}+H_{1}+\ldots +H_{T}$$

(7) $$\left\{ \begin{array}{c} H_{1}=-\sum_{i=0}^{s_{1}}\sum_{j=0}^{t_{1}}\frac{P_{i,j}}{\omega_{0}}\ln\left(\frac{P_{i,j}}{\omega_{0}}\right),\\ \omega_{0}=\sum_{i=0}^{s_{1}}\sum_{i=0}^{t_{1}}P_{i,j}\\ H_{2}=-\sum_{i=s_{1}+1}^{s_{2}}\sum_{j=t_{1}+1}^{t_{2}}\frac{P_{i,j}}{\omega_{1}}\ln\left(\frac{P_{i,j}}{\omega_{1}}\right),\\ \omega_{1}=\sum_{i=s_{1}+1}^{s_{2}}\sum_{j=t_{1}+1}^{t_{2}}P_{i,j}\\ H_{T}=-\sum_{i=s_{T-1}}^{s_{T}}\sum_{j=t_{T-1}}^{t_{T}}\frac{P_{i,j}}{\omega_{T}}\ln\left(\frac{P_{i,j}}{\omega_{T}}\right),\\ \omega_{T}=\sum_{i=s_{T-1}}^{s_{T}}\sum_{j=t_{T-1}}^{t_{T}}P_{i,j} \end{array}\right.$$

where *H_i_* denotes the entropy of the *i-th* image segmentation. {ω_1_, ω_2_, …, ω_*T*_} are the sum of the grayscale levels in the threshold interval.

### Ant colony algorithm for continuous domains

The original ACO algorithm could only deal with discrete optimization problems. Socha and Dorigo (2008) extended ACO to a continuous domain (ACOR). ACOR has excellent performance and has great potential for improvement. Meanwhile, the ACOR has a robust theoretical framework. Therefore, this study optimizes the optimal threshold based on ACOR. The traditional ACOR is as follows.

The archive mechanism is a significant feature of ACOR. The essence of the archive of solutions mechanism is to simulate the pheromone model. The total number of individuals is *N*, dimension is *dim*. The population is denoted as *X* = {*X*_1_, …, *X*_*k*_}, and the *i-th* individual is denoted as $X_{i}=\left(x_{i}^{1},\ldots x_{i}^{dim}\right),i\in \left[1,N\right]$. The size of the solution archive is set to *k*. At each iteration, the mechanism keeps *k* solutions with the best fitness values from *k* + *N* search individuals (the *k* best search agents in the previous iteration and the *N* search agents in the population of the current iteration), and stores them in the solution archive. The solution archive is denoted by S = {*S*_1_, …, *S*_*k*_}, and individuals in the solution archive are represented as $S_{m}=\left(s_{m}^{1},\ldots s_{m}^{dim}\right),m\in \left[1,k\right]$. In the archive, each search agent *S_m_* corresponds to a weighting factor ω_*m*_ and a probability *p_m_*. ω_*m*_ and *p_m_* are calculated from Eqs. 8, 9, respectively.


(8) $$\omega_{m}=\frac{1}{qk\sqrt{2\pi }}\text{exp}\left[-\frac{{\left(m-1\right)}^{2}}{2q^{2}k^{2}}\right]$$

(9) $$p_{m}=\frac{\omega_{m}}{\sum_{r=1}^{k}\omega_{r}}$$

where *q* is a parameter that is used to reconcile the local search with the global search.

The guide solution *S_g_* is selected from the solution archive based on the roulette selection method and probability *P*. The μ and σ required to update the population can be derived from the information on each dimension of *S_g_* and *S*. μ and σ are calculated from Eqs. 10–12, respectively.


(10) $$\mu_{j}=s_{g}^{j}$$

(11) $$\sigma_{j}=\xi D_{j},j=1,\ldots ,dim$$

(12) $$D_{j}=\sum_{r=1}^{k}\frac{\left|s_{r}^{j}-s_{g}^{j}\right|}{k-1}$$

where *ξ* is used to replace the pheromone evaporation rate. *D_j_* is the Manhattan distance. $\left|s_{r}^{j}-s_{g}^{j}\right|$ denotes the distance from each solution to the reference individual in the archive.

The update formula for the current search agent *X_i_* is shown in Eq. 13.


(13) $$x_{i}^{j}=normrnd\left(\mu_{j},\sigma_{j}\right)$$

where *normrnd*(μ, σ) is the normal distribution function, μ denotes the mean vector, and σ denotes the standard deviation vector.

## Enhanced ant colony optimization for continuous domains-based segmentation model

This section details the proposed EACOR and the EACOR-based thresholding image segmentation model.

### The proposed enhanced ant colony optimization for continuous domains

#### Motivation

In order to increase the convergence speed while improving the accuracy. This study introduced the soft besiege strategy. The soft besiege strategy is inspired by HHO (Heidari et al., 2019b). The soft besiege strategy ensures a more reasonable ratio of global exploration and local exploitation of the algorithm, which is conducive to searching for higher quality solution. At the beginning of the optimization process, the algorithm tends to explore the entire search space more globally, which accelerates convergence and reduces the local optimum risk. Toward the end of the optimization process, the algorithm focuses more on further exploitation around the current optimal solution, improving the convergence accuracy of the algorithm. The pursuit strategy can enhance the local exploitation performance of the algorithm.

#### The soft besiege strategy

**FIGURE 2:**
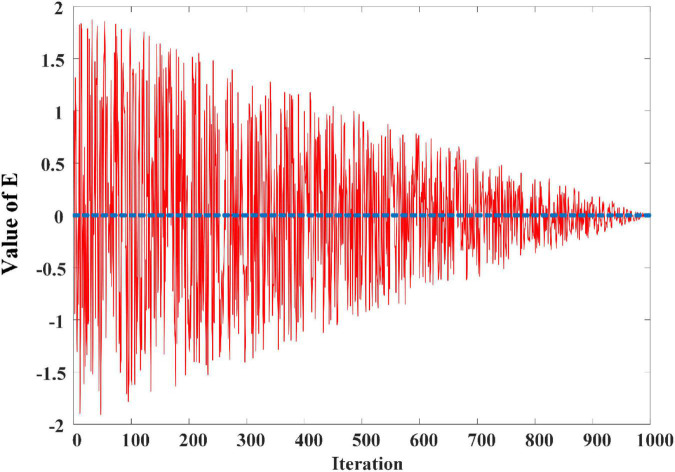
The change curve of **E** in 1000 iterations based on Heidari et al. (2019b).

The adaptive step size facilitates to coordinate the exploration and exploitation of the proposed method. The exploration phase enables the search agents to conduct a global search with a big step size. In the exploitation phase, the search agent performs the local search with a small step size. *E* is calculated from Eq. 14.


(14) $$E=2\left(1-\frac{FEs}{MaxFEs}\right)\left(2r_{1}-1\right)$$

where *FEs* is the number of current function evaluation. *MaxFEs* is the maximum number of function evaluation. *r_1_* is a randomly generated number between 0 and 1. From Eq. 14, as the number of iterations rises, we know that |*E*| drops from 2 to 0. The variation of *E* with the increasing number of iterations is shown in Figure 2.

The soft besiege strategy uses the average position of all ants in the colony, the random ant position, and the food position (global optimal solution) to influence the current ant movement. The soft besiege strategy consists of two phases according to the optimization process.

When the current number of iterations is 1, the update formula for the current individual $X_{i}=\left(x_{i}^{1},\ldots x_{i}^{dim}\right),i\in \left[1,N\right]$ is shown in Eq. 15.


(15) $$x_{i}^{j}=\left\{ \begin{array}{c} x_{rand}^{j}-r_{2}\times \left|x_{rand}^{j}-2r_{3}\times x_{i}^{j}\right|,r_{6}>0.5\\ \left(x_{best}^{j}-x_{mean}^{j}\right)-r_{4}\left(r_{5}\left(ub_{j}-lb_{j}\right)+lb_{j}\right),r_{6}\leq 0.5 \end{array}\right.$$

where $x_{rand}^{j}$, $x_{best}^{j}$, and $x_{mean}^{j}$ denote the *j-th* component of the random individual, optimal individual and average individual in the population, respectively. *r*_2_, *r*_3_, *r*_4_, *r*_5_, and *r*_6_ are randomly generated between 0 and 1. *ub*_*j*_ and *lb*_*j*_ denote the maximum and minimum values of the *j-th* component, respectively. When *r_6_* > 0.5, $x_{i}^{j}$ is updated according to the position of other individual. When *r*_6_ ≤ 0.5, $x_{i}^{j}$ updates its position based on the current best individual ant and the mean value of the population. This stage ensures that the colony can explore the search space more extensively and find the global optimal solution more easily.

When the number of iterations is greater than one, the main factor affecting the update of individual position is the current optimal individual. In addition, to improve the convergence accuracy and prevent falling into local optimum, random step *J* and Lévy flight are introduced. The updated formula for *J* is shown in Eq. 16.


(16) $$J=2\left(1-r_{7}\right)$$

where *r_7_* means random numbers between 0 and 1.

*J* and Lévy flight ensures that an individual can search locally at the current position and jump out of the local optimum by the feature of Lévy flight between long and short steps. At the next iteration, the individual *X*_*temp*_ that may replace the current *X_i_* is generated by Eqs. 17, 18.


$$x_{temp}^{j}=\left\{ \begin{array}{c} \left(x_{best}^{j}-x_{i}^{j}\right)-E\left|J\times x_{best}^{j}-x_{i}^{j}\right|,r_{8}\geq 0.5\;\left(17\right)\\ x_{best}^{j}-E\left|J\times x_{best}^{j}-x_{i}^{j}\right|,r_{8}<0.5\;\left(18\right) \end{array}\right.$$

where $x_{temp}^{j}$ denotes the *j-th* component of *X*_*temp*_. *r_8_* is randomly generated between 0 and 1.

In addition, when *r*_8_ < 0.5, the current individual’s position is further developed based on local information to obtain a better solution. If the fitness value of *X*_*temp*_ is better, the next search step is performed according to Eq. 19. Otherwise, the original position is retained.


(19) $$x_{temp}^{j}=x_{best}^{j}-E\left|J\times x_{best}^{j}-x_{i}^{j}\right|+r_{9}\times Levy$$

where *Levy* denotes Lévy flight. *r_9_* denotes a random number between 0 and 1.

As a result, the individual with the best fitness value of *X*_*temp*_ and *X_i_* is retained for the next iteration based on the current individual. The pseudo-code of the soft besiege strategy is shown in Algorithm 1.

**Algorithm 1:** Pseudo-code of the soft besiege strategy.

**Input:** *N, dim, ub, lb, MaxFEs*, object function *fobj*, current iteration number *t*, population *X*(*t*) = {*X*_1_, …, *X*_*N*_}. **Output:** Updated population *X*(*t* + 1) = {*X*_1_, …, *X*_*N*_} and its function value. Find the optimal individual *X*_*best*_ according to the function value; **For** *i* = 1: *N* Calculate *E* according to Eq. 14; **For** *j* = 1: *dim* **If** *t* equals 1 Update current individual $x_{i}^{j}$ by Eq. 15; **Else then** Update *J* by Eq. 16; **If** *r*_8_ ≥ 0.5 Update the individual $x_{temp}^{j}$ by Eq. 17; **Else then** Update the individual $x_{temp}^{j}$ by Eq. 18; **If***fobj*(*X*_*temp*_) better than *fobj*(*X*_*i*_) Update the individual $x_{temp}^{j}$ by Eq. 19; **End** **End** **End** Update *X_i_* according to the fitness function values of *X*_*temp*_ and *X_i_*; **End** **End**

#### The chase strategy

The chase strategy mainly updates the current individual’s position based on the guided individual $S_{g}=\left(s_{g}^{1},\ldots s_{g}^{dim}\right)$ and the global optimal *X*_*best*_. Since the two best individuals influence the current position, the strategy gradually devotes more computing resources to local location exploitation. The chase strategy is divided into two cases as shown in Eq. 20, by comparing the function value of *S_g_* and *X_i_*.


(20) $$x_{temp}^{j}=\left\{ \begin{array}{c} s_{g}^{j}+r_{10}\left(s_{g}^{j}-x_{i}^{j}\right)+r_{11}\left(x_{best}^{j}-s_{g}^{j}\right),\\ fobj\left(S_{g}\right)\mathrm{better}\mathrm{than}\mathrm{fobj}\left(X_{i}\right)\\ x_{i}^{j}+r_{12}\left(x_{i}^{j}-s_{g}^{j}\right)+r_{13}\left(x_{best}^{j}-x_{i}^{j}\right),otherwise \end{array}\right.$$

where *r*_10_, *r*_11_, *r*_12_, *r*_13_ denote the random numbers between 0 and 1. The pseudo-code of the chase strategy is shown in Algorithm 2.

**Algorithm 2:** Pseudo-code of the chase strategy.

**Input:** *N, dim*, object function *fobj*, population *X*(*t*) = {*X*_1_, …, *X*_*N*_}. **Output:** Updated population X(*t* + 1) = {*X*_1_, …, *X*_*N*_} and its function value. Find the optimal individual *X*_*best*_ according to the function value; **For** *i* = 1: *N* **For** *j* = 1: *dim* Update the individual $x_{temp}^{j}$ by Eq. 20; **End** Update *X_i_* according to the fitness function values of *X*_*temp*_ and *X_i_*; **End**

#### Implementation of enhanced ant colony optimization for continuous domains

**FIGURE 3:**
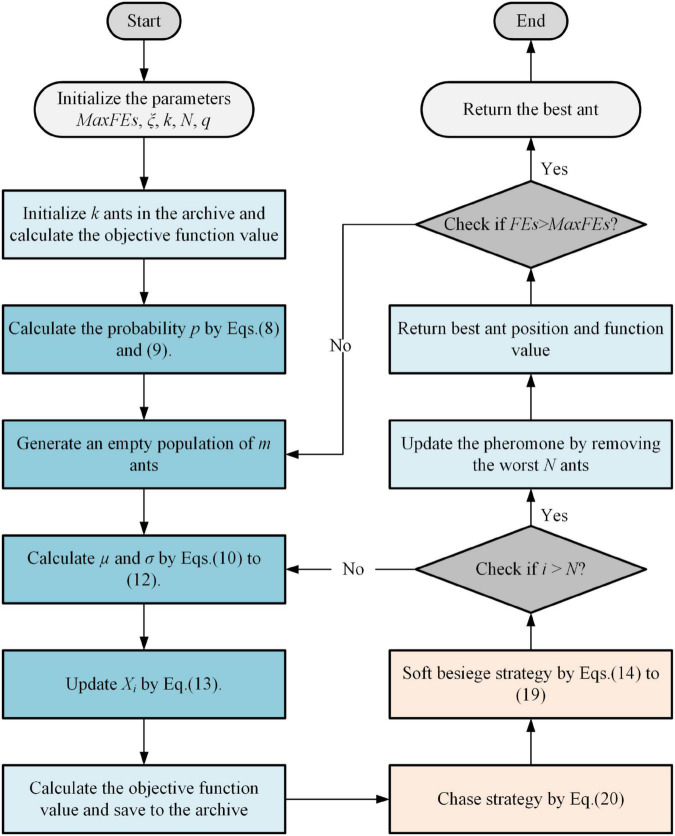
Flowchart of EACOR.

This subsection describes the process of implementing EACOR. Step 1: the parameters of the algorithm are defined. Step 2: the archive is initialized and the fitness value is calculated. Step 3: ACOR’s core update formula of is executed. Step 4: the chase strategy is used to further exploit the population position. Step 5: update the population according to the soft besiege strategy. Step 6: remove *N* bad solutions from *k* + *N* individuals in this iteration. Step 7: the optimal individuals are output. The flow chart of EACOR is shown in Figure 3.

### Implementation of the image segmentation model

In this study, a multi-level threshold segmentation method based on EACOR and Kapur’s entropy is used to achieve high-quality segmentation of melanoma images. According to the above method description, multi-threshold image segmentation is achieved according to the following steps.

Step 1: The original image is transformed into grayscale and NLM filtering images.

Step 2: The grayscale and NLM filtering images are mapped in an NLM two-dimensional histogram.

**FIGURE 4:**
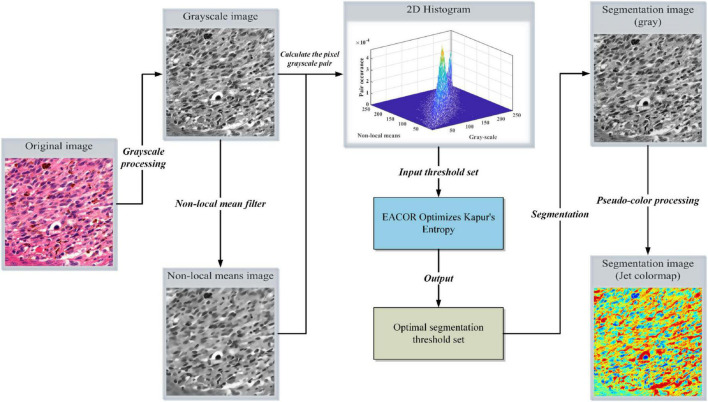
Flowchart of image segmentation.

Step 3: Kapur’s entropy is used as the fitness function for the image information. According to the observation in Figure 4, the most useful image information is distributed in the sub-regions of the main diagonal of the 2D histogram, so we only compute these regions. The objective function is optimized using the EACOR algorithm proposed in this paper. The final optimal value is used as the optimal threshold for this image.

Step 4: The segmented gray image and the color jet colormap image are obtained according to the optimal solution information.

The details of the proposed segmentation model are shown in Figure 4.

## Experiments and results analysis

In this section, the algorithm’s performance was verified in two main aspects: the benchmark function experiment and the image segmentation experiment. For the benchmark function experiments, all experiments were tested on the IEEE CEC2014 benchmark function. In addition, the global optimization performance of EACOR is verified by objective comparison experiments with some advanced methods. The EACOR-based multi-threshold image segmentation method segmented nine melanomas at different thresholds for the image segmentation experiments. Three segmentation quality assessment criteria were used to test the above results. To test whether the obtained data were statistically significant, the Wilcoxon signed-rank test (WSRT) (Garcia et al., 2010) and the Friedman test (FT) (Derrac et al., 2011) were used as statistical criteria for the data in this paper.

It is worth noting that ‘+,’ ‘−,’ and ‘=’ results appear in the comparison between the baseline function experiment and the image segmentation experiment. ‘+’ Indicates that the difference between the two results is significant and that the results of the proposed method are better. ‘−’ Indicates that the difference between the two results is significant and that the proposed method results are worse. ‘=’ Means that the difference between the two results is insignificant, and the performance of the two algorithms can be considered similar.

Moreover, all experiments were performed on a computer with Intel(R) Xeon(R) CPU E5-2620 v4 @ 2.10GHz processor and 16GB RAM, using MATLAB R2018b.

### Experiments on benchmark functions

#### Experimental conditions and environmental settings

The benchmark function experiments in subsection “Experiments on benchmark functions” focused on testing the global optimization performance of EACOR. IEEE CEC2014 test suite consists of unimodal, multimodal, hybrid, and composition functions that effectively validate the exploration and exploitation capabilities of the algorithms. Details of all IEEE CEC2014 benchmark functions can be referred to Liang et al. (2013). In order to ensure the fairness of the algorithm, all common parameters were standardized, as shown in Table 1. In addition, Table 2 shows the average ranking of the key parameters tested on the 30 functions. It was finally determined that *ξ* was set to 1, *k* was set to 10, and *q* was set to 0.5.

**TABLE 1:** Details of the public parameters.

Name	Remark	Value
*N*	The population size	30
*dim*	Objective function dimension	30
*MaxFEs*	The maximum number of evaluations	300,000
*ub*	Maximum value available in the search space	100
*lb*	Minimum value available in the search space	−100
*Flod*	Number of independent experiments	30

**TABLE 2:** Values of important parameters in EACOR.

*ξ*	Average rank	*k*	Average rank	*q*	Average rank
*ξ*(0.5)	2.87	*k*(5)	3.17	*q*(0.1)	4.17
*ξ*(0.75)	2.73	*k*(8)	3.13	*q*(0.3)	3.30
*ξ*(1)	**2.40**	*k*(10)	**2.60**	*q*(0.5)	**2.40**
*ξ*(1.25)	2.70	*k*(12)	2.93	*q*(0.7)	2.50
*ξ*(1.5)	2.97	*k*(15)	3.17	*q*(0.9)	2.63

In addition, bold in the table indicates the best data.

#### The strategy combination comparison test

Enhanced ant colony optimization for continuous domains introduced the soft besiege strategy and the chase strategy. In order to verify whether the two enhanced strategies can improve EACOR’s optimization capabilities, a strategy comparison experiment was set up in this subsection. The four combinations of the two strategies are shown in Table 3, where ‘1’ means that the strategy was used and ‘0’ means that the strategy was not used.

**TABLE 3:** Four combinations of two strategies in EACOR.

Methods	Soft besiege strategy	Chase strategy
EACOR	1	1
ACOR_S	1	0
ACOR_C	0	1
ACOR	0	0

Table 4 shows the rankings of WSRT and FT and the comparison results. For WSRT, EACOR ranked the best of the four algorithms with an average ranking of 1.93 on the 30 benchmark functions. For FT, EACOR ranked first, and its average ranking was 1.98. In the last column of Table 4, EACOR had six results that were better than ACOR_S, four results that were worse than ACOR_S, and other results that were like ACOR_S. Compared to ACOR without both strategies, EACOR won in 27 functions and failed in only 2 functions.

**TABLE 4:** Comparative results of strategy combination comparison experiment.

Methods	WSRT	FT	+/−/=
EACOR	**1 (1.93)**	**1 (1.98)**	**∼**
ACOR_S	2 (2.07)	2 (2.07)	6/4/20
ACOR_C	3 (2.14)	3 (2.21)	9/2/19
ACOR	4 (3.74)	4 (3.76)	27/2/1

In addition, bold in the table indicates the best data.

**FIGURE 5:**
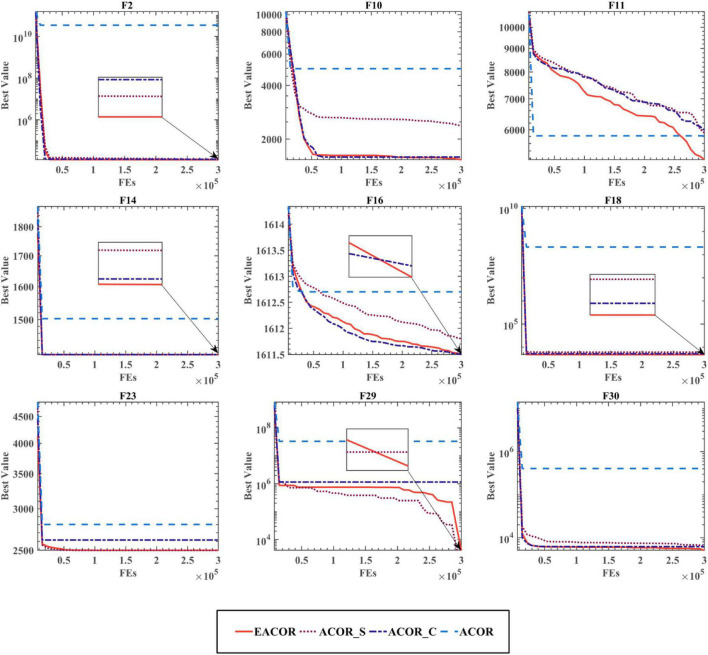
The convergence curve of the EACOR strategy combination.

The convergence curves for the four different combinations of the two techniques are shown in Figure 5 and Supplementary Figures B.1, B.2. From the convergence curves, we can see that EACOR has better convergence accuracy than the other three algorithms. Furthermore, it can be seen at F11, F29, and F30 that there was a rapid downward trend in the curves of EACOR and ACOR_S for evaluation numbers between 250,000 and 300,000. It is due to the process of EACOR escaping from the local optimum solution when dealing with some multi-peaked functions. It shows that the soft besiege strategy introduced by the algorithm improved the ability of the algorithm to avoid falling into the local optimum solution.

According to the above analysis, only the ACOR algorithm with both the soft besiege strategy and the chase strategy can reach the optimal state.

#### The qualitative analysis

**FIGURE 6:**
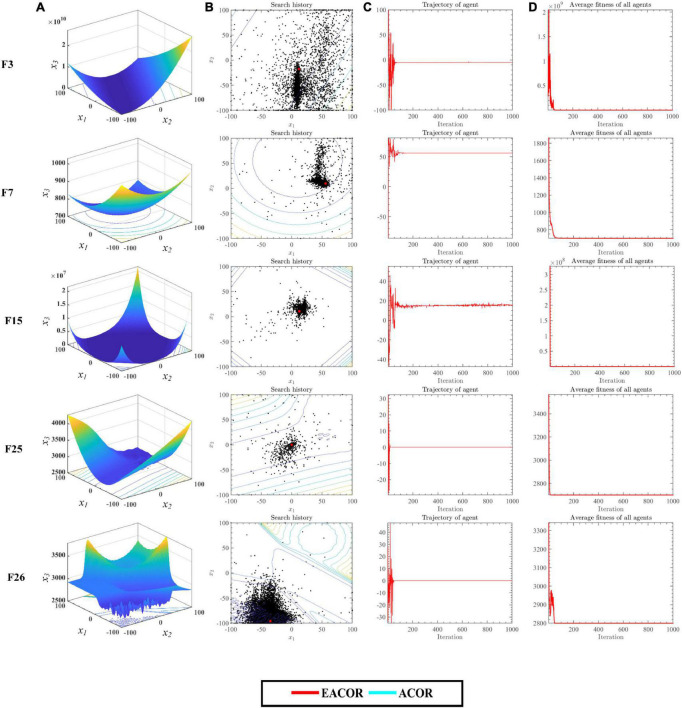
**(A)** Three-dimensional location distribution of EACOR. **(B)** Distribution of historical search tracks of EACOR. **(C)** Historical trajectory of EACOR’s component. **(D)** Average fitness of EACOR.

To analyze the impact of the introduced strategy on ACOR, the search process of EACOR and ACOR was analyzed through 1000 iterations. Figure 6 shows the search process of some functions of EACOR on IEEE CEC2014. Figure 6A shows the 3-D search space of the global optimization problems. Figure 6B shows all exploration trajectories in the search space, where the red dot indicates the optimal positions. Figure 6C depicts the algorithm’s search trajectory in the first dimension. EACOR’s average fitness value is seen in Figure 6D.

Figure 6B demonstrates that most search agents focus their efforts on a local search around the global optimal solution, whereas only a tiny number of search agents conduct a global search for the optimal solution. This shows that EACOR has not only local but also global search capability. Figure 6C shows that EACOR has a sharp oscillation in the search trajectory curve in the early stage of the search, and then the curve becomes smooth. This change ensures that the algorithm improves both the convergence accuracy and the speed of convergence. Figure 6D shows that EACOR has a variety of search agents during the preliminary iterations. The diversity of search agents, on the other hand, decreases as the number of iterations increases. This confirms the transformation of the algorithm from the exploration phase to the exploitation phase.

**FIGURE 7:**
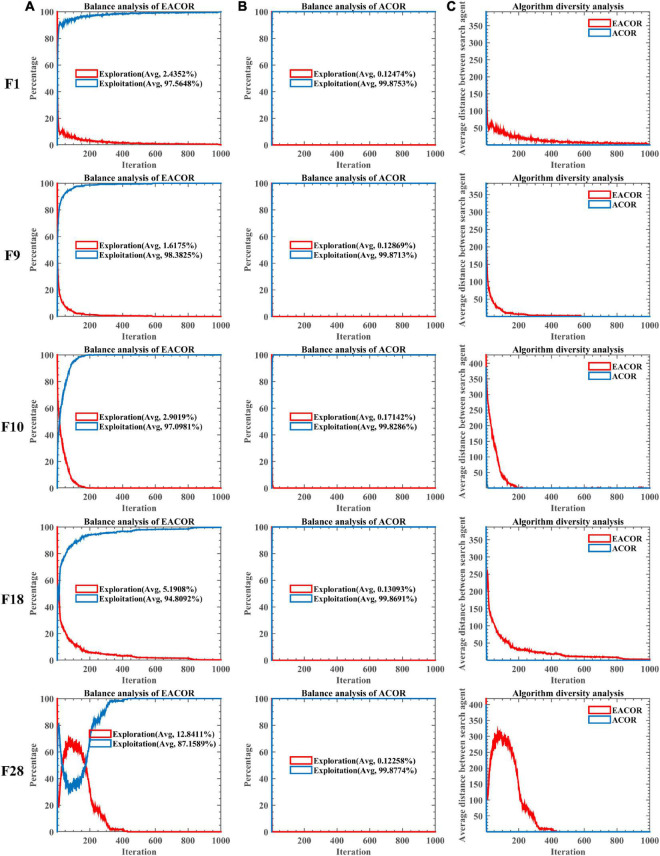
**(A)** The balance tests of EACOR. **(B)** The balance tests of ACOR. **(C)** The diversity tests of EACOR and ACOR.

To further analyze how the introduction mechanism improves the ACOR search capability. We have conducted balanced experiments on the Exploration phase and Exploitation phase of EACOR and ACOR based on 30 functions of IEEE CEC2014. In addition, experiments on the diversity of the two algorithms were conducted. The experimental result is shown in Figure 7 and Supplementary Figures B.3–B.7. The red and blue lines in Figures 7A,B represent the Exploration and Exploitation phases, respectively. The first and second column images show the balance test results for EACOR and ACOR, respectively. From the first two columns of test results, it can be seen that the exploitation phase of EACOR and ACOR dominates and facilitates the algorithm to explore the known solutions further. By comparing the exploration and exploitation phases of the two methods, it can be seen that EACOR enhances the algorithm’s exploration of the search space and improves the probability of obtaining an optimal solution. A comparison of Figures 7A,B in the same row shows that the search ratio of EACOR in the Exploration phase is significantly higher than that of ACOR. This leads to the situation shown in Figure 7C, where the population diversity of EACOR is significantly larger than that of ACOR at the beginning of the iteration and gradually decreases at the end of the iteration. From the above analysis, we can conclude that EACOR can jump out of the local optimal solution and achieve better convergence accuracy because the global search capability is stronger than that of ACOR.

#### The stability experiment of enhanced ant colony optimization for continuous domains

To verify the effect of the real problem dimension component on the EACOR optimization performance (Zhu B. et al., 2018), this subsection analyzed the experimental results of the EACOR algorithm for 30 benchmark functions in high dimensions (*dim* was set to 50 and 100).

Table 5 shows the ranking of EACOR and ACOR for WSRT and FT in dealing with high dimensional problems and the comparison results. From the table, EACOR was the best in both the WSRT and FT statistical tests in both 50 and 100 dimensions. Furthermore, EACOR achieved stronger optimization results than ACOR for 26 functions in both dimensions, indicating that EACOR can still show excellent optimization performance when dealing with high-dimensional complex problems.

**TABLE 5:** Comparative results of stability analysis.

	dim = 50		dim = 100	
	EACOR	ACOR	EACOR	ACOR
WSRT	**1 (1.10)**	2 (1.90)	**1 (1.13)**	2 (1.87)
FT	**1 (1.10)**	2 (1.90)	**1 (1.11)**	2 (1.89)
+/−/=	**∼**	26/2/2	**∼**	26/3/1

In addition, bold in the table indicates the best data.

**FIGURE 8:**
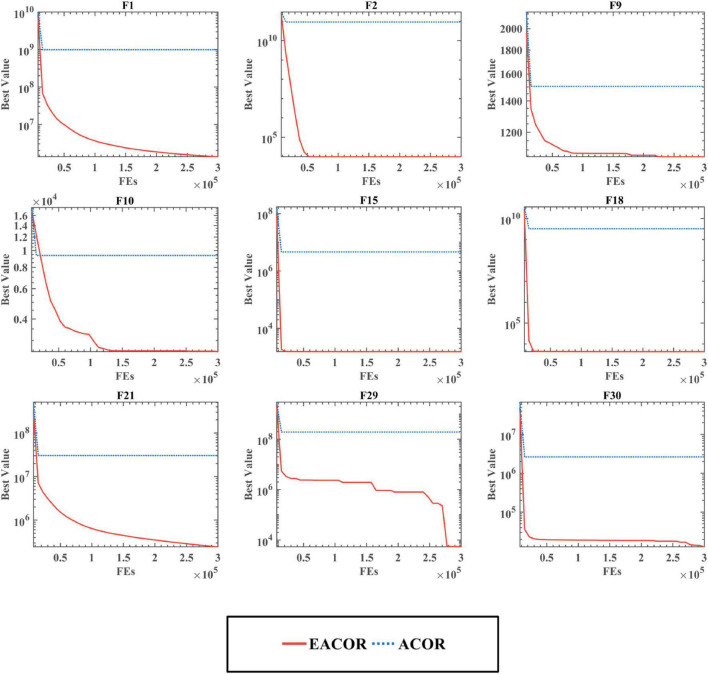
Convergence curves of EACOR and ACOR at 50 dimensions.

**FIGURE 9:**
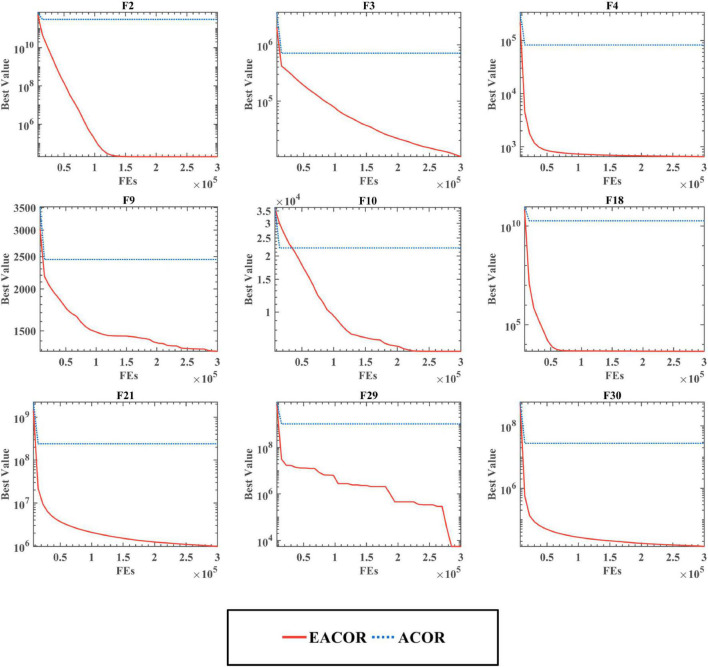
Convergence curves of EACOR and ACOR at 100 dimensions.

In addition, Figures 8, 9 show the convergence curve of the algorithm in dealing with high-dimensional problems. Although the algorithm’s convergence accuracy was reduced due to the increase in problem dimensionality, EACOR was less affected than the original algorithm. It is worth noting that the convergence curves for F9, F10, F29, and F30 can be observed; EACOR can still jump out of the local optimal solution at the late stage of the iteration in the high dimension. As a result, EACOR was superior in terms of convergence performance and escape from local optimum solutions.

#### Comparison experiment with the original algorithms

To enable a more objective evaluation of EACOR’s performance, it was compared to ten well-known competitors in this subsection. These algorithms included ACOR, DE, FA, GWO, WOA, HHO, MFO, SCA, SFS, and TSA.

Table 6 shows the average ranking of the optimization results for the 30 benchmark functions. Among the 11 methods, EACOR ranked first in both WSRT and FT, with an average ranking of 2.37 and 2.77, respectively. The suboptimal method is DE, which has an average ranking of 2.97 and 3.2 for WSRT and FT, respectively. Furthermore, EACOR can beat DE on 18 functions, while DE outperformed EACOR on only 10 functions, and the other two results were considered equal. It is noteworthy that the worst-performing method of both statistical methods was ACOR, which shows that the improvement strategy proposed in this study can adequately improve the algorithm’s performance.

**TABLE 6:** Comparative results of EACOR with ten original algorithms.

Methods	WSRT		FT		+/−/=
	Avg.	Rank	Avg.	Rank	
EACOR	**2.37**	**1**	**2.77**	**1**	**∼**
ACOR	9.53	11	9.31	11	26/2/2
DE	2.97	2	3.2	2	18/10/2
FA	9.37	10	9.21	10	27/1/2
GWO	5.17	6	5.14	5	23/3/4
HHO	4.03	3	4.28	3	18/3/9
MFO	7.2	8	6.96	8	26/3/1
SCA	8.7	9	8.71	9	27/1/2
WOA	6.63	7	6.43	7	25/3/2
SFS	4.67	4	4.82	4	20/3/7
TSA	4.97	5	5.19	6	24/3/3

In addition, bold in the table indicates the best data.

**FIGURE 10:**
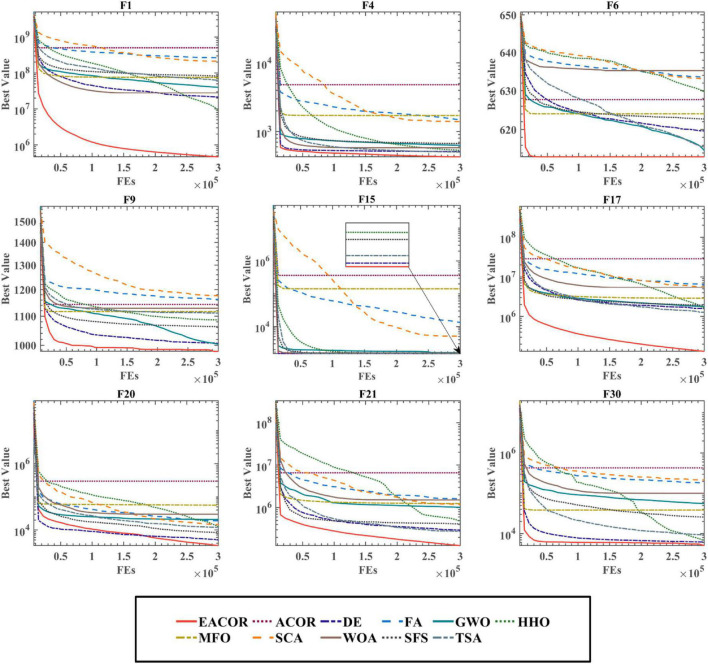
Convergence curves of EACOR with ten original algorithms.

Figure 10 and Supplementary Figures B.12, B.13 show the convergence of the 11 algorithms on 30 benchmark functions. We can see that EACOR is at the bottom of the convergence curve. In F1, F6, F9, F21, F30, and EACOR finds better solutions and converges faster than the other algorithms. In F1, F9, F17, F20, F21, F30, and EACOR still finds better solutions at the end of the iterations, especially in F21, EACOR converges at the beginning of the iteration with accuracy second to that of DE, but EACOR’s advantage of jumping out of the local optimum at the end of the iteration makes the result better than DE’s result.

The means and standard deviations of 30 independent runs of the experiments indicate that the algorithm can obtain excellent and stable optimization results. WSRT and FT demonstrate the statistical significance of the experimental results. Overall, EACOR is more competitive than some advanced and original algorithms.

#### Comparison experiments with the peers

To further demonstrate the superiority of the EACOR algorithm in terms of performance, EACOR was compared with ten other excellent improved algorithms. These peers were CDLOBA, CBA, HGWO, ASCA_PSO, SCADE, m_SCA, IGWO, OBLGWO, ACWOA, and BMWOA.

Table 7 shows the ranking of EACOR with these ten peers for both statistical methods. EACOR performed best in WSRT and FT, with an average ranking of 2.17 and 2.62, respectively. In addition, EACOR can beat the second-ranked IGWO on 23 functions and be disadvantaged on only 4 functions. The various comparisons in Table 7 show that EACOR has better optimization performance and can handle different optimization problems better.

**TABLE 7:** Comparative results of EACOR with ten peers.

Methods	WSRT		FT		+/−/=
	Avg.	Rank	Avg.	Rank	
EACOR	**2.17**	**1**	**2.62**	**1**	**∼**
CDLOBA	6.67	8	6.31	7	22/3/5
CBA	5.93	4	5.71	4	21/3/6
HGWO	7.57	9	7.45	9	24/3/3
ASCA_PSO	6.33	6	6.19	6	26/1/3
SCADE	8.93	11	8.97	11	24/2/4
m_SCA	5.93	4	5.93	5	25/3/2
IGWO	3.73	2	4.12	2	23/4/3
OBLGWO	4.27	3	4.57	3	24/2/4
ACWOA	7.63	10	7.54	10	23/3/4
BMWOA	6.43	7	6.59	8	28/1/1

In addition, bold in the table indicates the best data.

**FIGURE 11:**
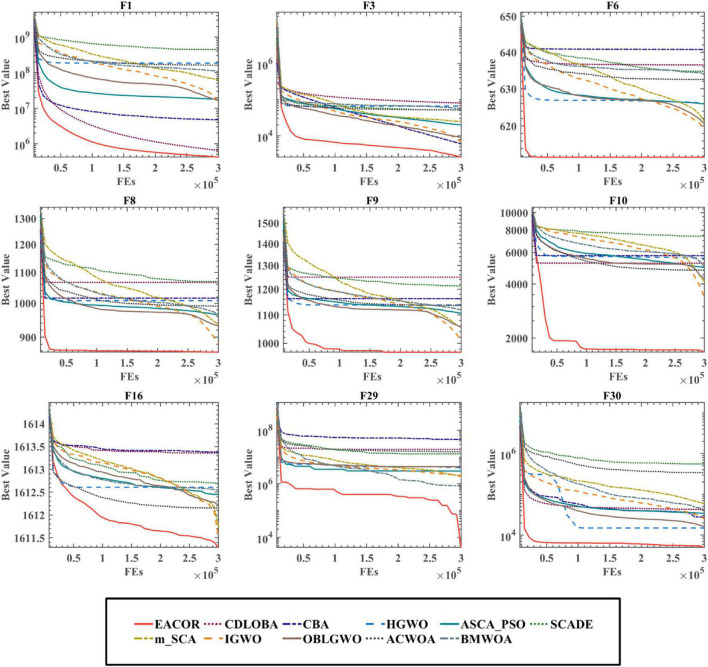
Convergence curves of EACOR with ten peers.

Figure 11 and Supplementary Figures B.14, B.15 show the convergence of EACOR with ten advanced peer methods on IEEE CEC2014, and it can be seen that EACOR can achieve better optimization results in most functions compared to other methods. In addition, by looking at F3, F16, and F29, we can see that the convergence curves of EACOR are still significantly skewed in the late iterations, which indicates that EACOR has an excellent ability to jump out of the local optimal solution.

Based on the above analysis, we can conclude that EACOR still has significant advantages not only compared with the original algorithm but also compared with the improved algorithm in terms of convergence accuracy, convergence speed, and prevention of premature convergence.

### Experiments on image segmentation

To validate the performance of the EACOR-based multi-level threshold image segmentation model proposed in this paper, we used image segmentation on nine real melanoma pathology images and compared them with some well-known algorithms. The original pathology images and the non-local mean two-dimensional histogram are shown in Supplementary Figure B.16. Notably, we conducted experiments at different thresholds to investigate the competitive performance of the proposed segmentation method at different thresholds. Then, the obtained segmentation results were analyzed.

#### Validation criteria for image quality

In order to judge the quality of the segmented image, three methods, PSNR, SSIM, and FSIM, were used to evaluate the segmentation results. Table 8 describes the evaluation methods PSNR, SSIM, and FSIM.

**TABLE 8:** Image quality evaluation index.

Name	Formula	Remark
PSNR (Huynh-Thu and Ghanbari, 2008)	*PSNR* = 10⋅*log*_10_(*peak*^2^)/*MSE*	The larger PSNR value between the two images indicates that the image has less distortion after compression.
FSIM (Zhang et al., 2011)	** $FSIM=\frac{\sum_{x\in \Omega }S_{L}\left(x\right)\cdot PC_{m}\left(x\right)}{\sum_{x\in \Omega }PC_{m}\left(x\right)}$ **	The larger the FSIM value obtained, the better the segmentation effect.
SSIM (Wang et al., 2014)	$SSIM=\frac{\left(2\mu_{I}\mu_{K}+C_{1}\right)\left(2\sigma_{IK}-C_{2}\right)}{\left(\mu_{I}^{2}+\mu_{K}^{2}+C_{1}\right)\left(\sigma_{I}^{2}+\sigma_{K}^{2}+C_{2}\right)}$	The larger the SSIM value between two images, the smaller the image distortion, and its value range is [0, 1].

After obtaining the above three criteria, the evaluation data were statistically analyzed using the mean, standard deviation, WSRT, and FT. The final evaluation data of the image segmentation effect were obtained.

#### Melanoma image segmentation experiment

Since melanoma can be easily confused with a pigmented nevus, there are some mistakes in the process of pathological image detection. To segment melanoma effectively, we use the MTIS technique to segment melanoma pathological images. To see more clearly the value of EACOR in melanoma image segmentation, 9 pathological images of melanoma were segmented at 5 thresholds by EACOR with 9 similar algorithms, namely ACOR, CS, GWO, HHO, SCA, ACWOA, IGWO, m_SCA, and SCADE, according to the evaluation method in subsection “Validation criteria for image quality.”

**FIGURE 12:**
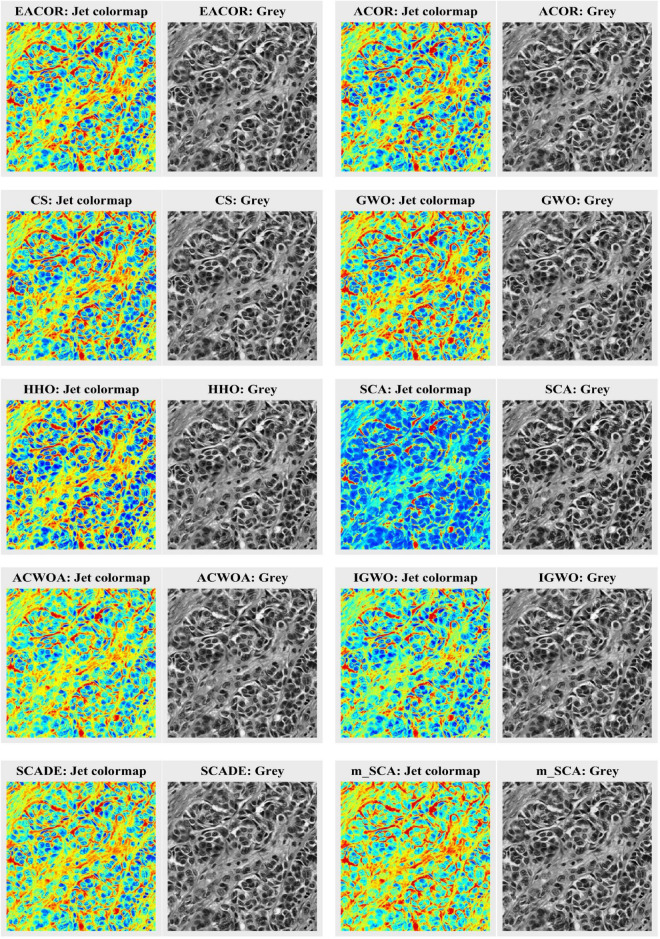
Segmentation results of all algorithms.

Figure 12 shows the jet colormap and gray images obtained after segmentation of nine melanoma images by each method. It is easy to observe that the image segmentation obtained by SCA and IGWO was inferior to the other methods. However, EACOR, ACOR, and CS were visually difficult to distinguish the segmentation quality. Therefore, in subsequent experiments, the performance of the methods was compared more visually through the three evaluation methods.

**FIGURE 13:**
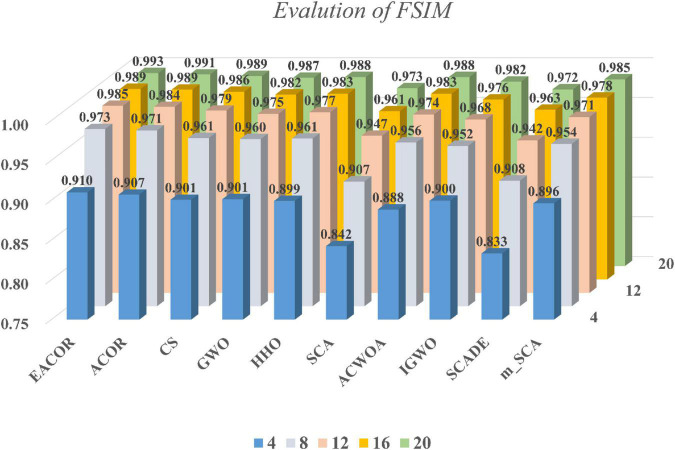
FSIM results at different thresholds for nine melanoma images.

**FIGURE 14:**
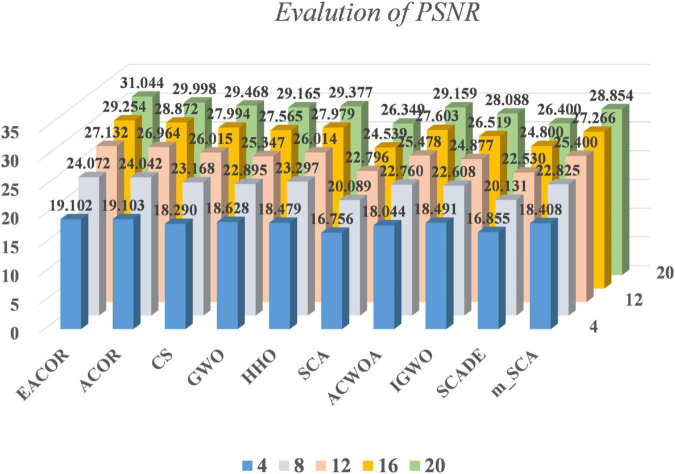
PSNR results at different thresholds for nine melanoma images.

**FIGURE 15:**
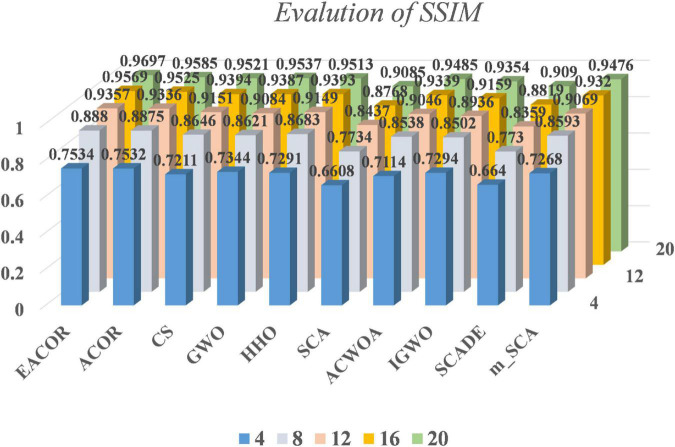
SSIM results at different thresholds for nine melanoma images.

**FIGURE 16:**
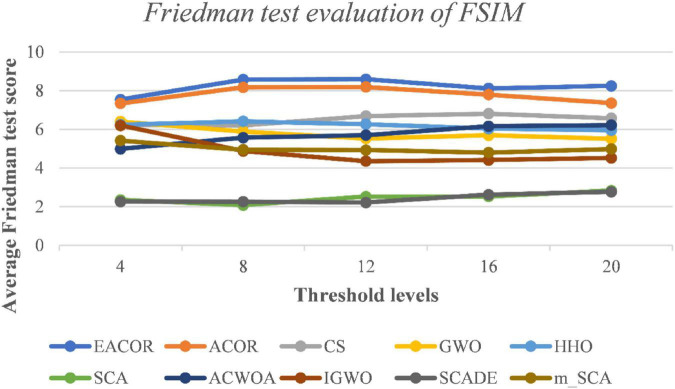
FT result of FSIM evaluation at different thresholds for nine melanoma images.

**FIGURE 17:**
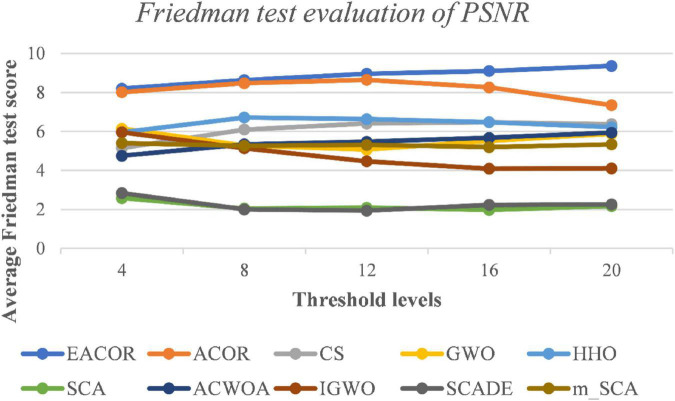
FT result of PSNR evaluation at different thresholds for nine melanoma images.

**FIGURE 18:**
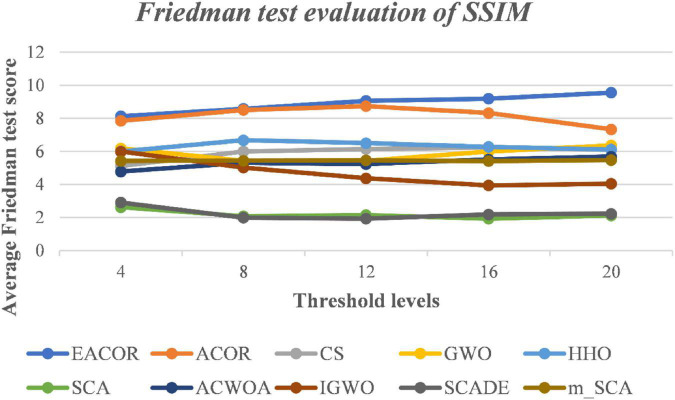
FT result of SSIM evaluation at different thresholds for nine melanoma images.

Supplementary Tables A.1–A.3 compare FSIM, PSNR, and SSIM at different thresholds. As can be seen from the table, EACOR ranked first for all thresholds. Furthermore, the difference between EACOR and ACOR for segmentation of 9 images was small at thresholds 4, 8, and 12. However, at 16 and 20 thresholds, most images segmented using EACOR were significantly better than those obtained with ACOR. Figures 13–15 show the three image evaluation metric scores at each threshold. The mean values of FSIM, PSNR, and SSIM were the highest for EACOR, indicating that the EACOR-based image segmentation method can achieve high-quality segmentation of melanoma images. And by comparing the thresholding results of the five levels of thresholding an increase in the threshold level between the experimentally set threshold levels is beneficial in improving the segmentation results. To further verify the significance of the obtained results, the experimental results were further analyzed by the Friedman test. Figures 16–18 show the FT results of the three evaluation criteria. The three FT results show that EACOR was the best compared to the other algorithms with the same conditions. The combination of FSIM, PSNR, and SSIM shows that the melanoma images obtained by this method retain more useful image features and have less image distortion.

**FIGURE 19:**
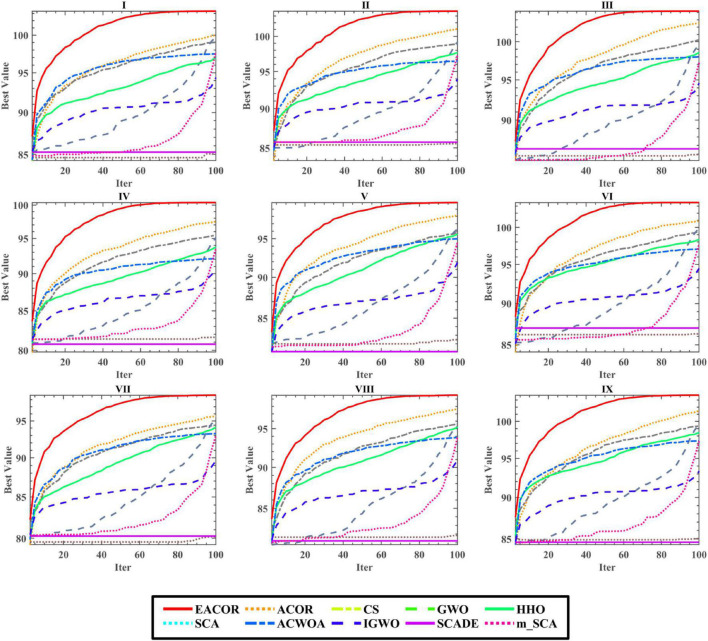
The convergence curve of 20-level threshold segmentation.

As the proposed image segmentation framework used Kapur’s entropy as the objective function of the segmentation threshold. Therefore, the larger Kapur’s entropy value indicates that the maximum amount of information is retained between the background and the target, which is more conducive to improving image segmentation quality. Supplementary Table A.4 shows that the maximum of Kapur’s entropy is obtained for different thresholds, and EACOR can still obtain the optimal Kapur’s entropy value in most cases. Figure 19 shows the convergence curves for each algorithm for nine images at 20 levels of thresholding. Based on the above analysis of Kapur’s entropy, the most reasonable solution was obtained by EACOR, followed by ACOR. Finally, Supplementary Figures B.17–B.25 show the optimal set of thresholds for the 9 images at 8 levels of thresholding.

## Discussion

We can draw the following conclusions based on the experimental results in subsection “Experiments on benchmark functions.” First, when EACOR is compared with ACOR in a higher dimension of the same function, the convergence of EACOR becomes faster, and the convergence accuracy is higher. It shows that EACOR has a better and more stable optimization performance than ACOR in different dimensions. Second, in analyzing the effect of the soft besiege strategy and the chase strategy on ACOR, the soft besiege strategy balances the exploration and development phases so that the global search ability of EACOR becomes weaker with increasing iterations. The local search ability becomes more robust with increasing iterations, effectively solving the problem of insufficient convergence accuracy of ACOR. Moreover, by observing the EACOR search process, we can conclude that EACOR can still perform the global search while performing the local search at the end. The chase strategy can increase the local search capability of EACOR. Thirdly, comparing EACOR with some peers and variants further demonstrates the strong optimization capability of EACOR. As a result, EACOR can cope with different complex optimization problems.

In subsection “Experiments on image segmentation,” the results of FSIM, PSNR, and SSIM are evaluated by analyzing 4, 8, 12, 16, and 20 thresholds. Then the convergence curves of mean, standard deviation, WSRT, FT, and optimal threshold combinations are used to verify the segmentation effectiveness of the EACOR-based multi-threshold segmentation method. Firstly, based on the results of 30 experimental means and standard deviations, it can be concluded that the experimental results are not coincidental, and EACOR shows excellent stability and segmentation ability. Secondly, based on the comparison results of WSRT and FT at 5 different thresholds, EACOR outperforms other segmentation methods at all thresholds, and as the threshold level increases, EACOR’s WSRT and FT results become better. This indicates that the higher the threshold value within a certain threshold range, the better EACOR’s segmentation results become. Thirdly, combining the optimal threshold combinations and the adaptation convergence curves of each method shows that EACOR can find the optimal threshold combination in the shortest time. The segmentation efficiency of the model is improved. It can be obtained through global optimization and image segmentation experiments that the EACOR-based multi-threshold Kapur’s entropy segmentation method is an excellent segmentation tool for melanoma images, which can provide samples with less redundant information for subsequent computer-aided diagnosis.

Because it is based on an improvement of the original algorithm, it inevitably makes the EACOR calculation more complex. The significant improvement in optimization performance makes the increased computational cost acceptable. Moreover, this problem can be overcome in subsequent work by introducing parallel computing techniques and high arithmetic devices. The superior optimization performance of EACOR ensures efficient image segmentation models, and provide greater possibilities for application to other fields in the future, such as disease prediction (Su et al., 2019; Li L. et al., 2021), recommender system (Li et al., 2014, 2017), information retrieval services (Wu et al., 2020a, 2021b), human activity recognition (Qiu et al., 2022), colorectal polyp region extraction (Hu K. et al., 2022), location-based services (Wu et al., 2020b, 2021a), text clustering (Guan et al., 2020), essay recommendation (Liang et al., 2021), image denoising (Zhang et al., 2020), drug-disease associations prediction (Cai et al., 2021), other disease image segmentation (Qi et al., 2022; Ren et al., 2022; Su et al., 2022), dynamic module detection (Ma et al., 2020; Li D. et al., 2021), drug discovery (Zhu F. et al., 2018; Li Y. et al., 2020), and road network planning (Huang et al., 2022).

## Conclusion and future works

To obtain higher quality segmentation results of pathological images in melanoma, this paper proposes a high-quality improvement algorithm EACOR based on ACOR. We also propose an MTIS method based on EACOR and Kapur’s entropy. EACOR introduces the soft besiege strategy and the chase strategy based on ACOR. In addition to addressing ACOR’s convergence speed and accuracy shortcomings, it enhances the ability of global search to keep the algorithm from falling into a local optimum. The following experiments were conducted to evaluate the usefulness of EACOR in practical applications. The first step is to assess EACOR’s ability to optimize its performance. On 30 benchmark functions from IEEE CEC2014, we tested EACOR and used the WSRT and the FT to analyze the results of our experiments statistically. We performed quantitative and qualitative analysis of the new strategy by a series of experiments, and the results showed that the soft besiege strategy and the chase strategy could enhance the optimization capability of EACOR. The stability of EACOR is demonstrated through high-dimensional experiments. To further validate the optimization performance of EACOR, we compare EACOR with 10 peers with excellent performance and 10 variants of the algorithm. The experimental results show that EACOR has the best optimization performance among these 20 similar algorithms. In the second step, we validate the segmentation effect of EACOR on melanoma images. For nine genuine melanoma pathology images, we used EACOR to conduct MTIS with EACOR. The NLM and two-dimensional histogram at the heart of MTIS is used in conjunction with the EACOR method to determine the best threshold for Kapur’s entropy. To fully demonstrate the segmentation capability of EACOR, we evaluate the segmentation results obtained by EACOR at 4, 8, 12, 16, 20 thresholds using FSIM, PSNR, SSIM as segmentation criteria. In the image segmentation experiments, we added 9 similar algorithms to compare with EACOR. The experimental results show that EACOR can perform effective MTIS for more complex melanoma images.

In future work, we will apply the powerful optimization capabilities of EACOR to other areas. For example, engineering optimization problems, feature selection, photovoltaic parameter identification and bankruptcy prediction are among the practical problems. In addition, the main area of EACOR is the segmentation of pathological images so we will use EACOR for more segmentation of melanoma pathological images. It is hoped that the quality of segmentation of pathological images from other diseases, including HE staining or immunohistochemical (IHC) staining can be applied to practice and improved.

## Data availability statement

The original contributions presented in this study are included in the article/Supplementary materials, further inquiries can be directed to the corresponding authors.

## Author contributions

XYa, XYe, AH, and ZX contributed to writing—original draft, writing—review and editing, software, visualization, and investigation. DZ, HC, and YL contributed to conceptualization, methodology, formal analysis, investigation, writing—review and editing, funding acquisition, and supervision. All authors contributed to the article and approved the submitted version.
