# Supplementary material for: Multi-threshold image segmentation for melanoma based on Kapur’s entropy using enhanced ant colony optimization
Source: Front Neuroinform. 2022 Nov 1;16:1041799. doi: 10.3389/fninf.2022.1041799 (PMC9663822; doi:10.3389/fninf.2022.1041799)
Supplement: Supplementary file 1 [file Data_Sheet_1.PDF]

Appendix A

Table A.1. The FSIM results of EACOR and similar algorithms on melanoma images.

Levels	Items	EACOR	ACOR	CS	GWO	HHO	SCA	ACWOA	IGWO	SCADE	m_SCA
4	Avg	**1.89**	2.33	4.11	4.00	5.11	9.33	7.44	4.89	9.67	6.22
	Rank	**1**	2	4	3	6	9	8	5	10	7
	+/ -/ =	~	1/0/8	5/0/4	5/0/4	5/0/4	9/0/0	9/0/0	4/0/5	9/0/0	7/0/2
8	Avg	**1.11**	2.00	4.11	4.44	4.22	9.67	6.22	7.00	9.33	6.89
	Rank	**1**	2	3	5	4	10	6	8	9	7
	+/ -/ =	~	0/0/9	9/0/0	9/0/0	7/0/2	9/0/0	8/0/1	9/0/0	9/0/0	9/0/0
12	Avg	**1.22**	1.89	3.44	5.11	4.44	9.00	5.78	7.33	10.00	6.78
	Rank	**1**	2	3	5	4	9	6	8	10	7
	+/ -/ =	~	0/0/9	6/0/3	9/0/0	8/0/1	9/0/0	7/0/2	9/0/0	9/0/0	9/0/0
16	Avg	**1.44**	1.67	3.56	5.00	5.11	9.67	4.78	7.67	9.33	6.78
	Rank	**1**	2	3	5	6	10	4	8	9	7
	+/ -/ =	~	3/0/6	6/0/3	9/0/0	6/0/3	9/0/0	6/0/3	9/0/0	9/0/0	9/0/0
20	Avg	**1.56**	2.22	3.44	5.00	5.11	9.44	4.44	7.78	9.56	6.44
	Rank	**1**	2	3	5	6	9	4	8	10	7
	+/ -/ =	~	5/0/4	5/0/4	7/0/2	8/0/1	9/0/0	6/0/3	9/0/0	9/0/0	9/0/0

Table A.2. The PSNR results of EACOR and similar algorithms on melanoma images

Levels	Items	EACOR	ACOR	CS	GWO	HHO	SCA	ACWOA	IGWO	SCADE	m_SCA

4	Avg	**1.44**	1.78	6.00	4.11	4.78	9.22	7.33	5.00	9.33	6.00
	Rank	**1**	2	6	3	4	9	8	5	10	6
	+/ -/ =	~	1/0/8	8/0/1	8/0/1	8/0/1	9/0/0	9/0/0	8/0/1	9/0/0	8/0/1
8	Avg	**1.44**	1.89	4.11	5.78	3.44	9.44	6.33	7.00	9.56	6.00
	Rank	**1**	2	4	5	3	9	7	8	10	6
	+/ -/ =	~	0/0/9	8/0/1	9/0/0	6/0/3	9/0/0	9/0/0	9/0/0	9/0/0	9/0/0
12	Avg	**1.11**	1.89	3.56	6.33	4.00	9.33	5.44	7.56	9.67	6.11
	Rank	**1**	2	3	7	4	9	5	8	10	6
	+/ -/ =	~	1/0/8	9/0/0	9/0/0	8/0/1	9/0/0	9/0/0	9/0/0	9/0/0	9/0/0
16	Avg	**1.00**	2.11	3.78	5.00	4.00	9.78	5.67	8.00	9.22	6.44
	Rank	**1**	2	3	5	4	10	6	8	9	7
	+/ -/ =	~	7/0/2	9/0/0	9/0/0	7/0/2	9/0/0	9/0/0	9/0/0	9/0/0	9/0/0
20	Avg	**1.00**	2.11	3.89	4.89	4.56	9.56	5.11	7.89	9.44	6.56
	Rank	**1**	2	3	5	4	10	6	8	9	7
	+/ -/ =	~	9/0/0	9/0/0	9/0/0	9/0/0	9/0/0	9/0/0	9/0/0	9/0/0	9/0/0

Table A.3. The SSIM results of EACOR and similar algorithms on melanoma images

Levels	Items	EACOR	ACOR	CS	GWO	HHO	SCA	ACWOA	IGWO	SCADE	m_SCA
4	Avg	**1.33**	1.78	6.00	4.33	4.78	9.11	7.33	5.00	9.33	6.00
	Rank	**1**	2	6	3	4	9	8	5	10	6
	+/ -/ =	~	1/0/8	8/0/1	7/0/2	6/0/3	9/0/0	9/0/0	8/0/1	9/0/0	8/0/1
8	Avg	**1.44**	1.78	4.44	5.44	3.56	9.44	6.56	7.00	9.56	5.78
	Rank	**1**	2	4	5	3	9	7	8	10	6
	+/ -/ =	~	0/0/9	8/0/1	9/0/0	6/0/3	9/0/0	8/0/1	9/0/0	9/0/0	9/0/0

12	Avg	**1.11**	1.89	3.78	5.56	4.22	9.33	6.00	7.56	9.67	5.89
	Rank	**1**	2	3	5	4	9	7	8	10	6
	+/ -/ =	~	1/0/8	9/0/0	9/0/0	8/0/1	9/0/0	9/0/0	9/0/0	9/0/0	9/0/0
16	Avg	**1.00**	2.00	4.22	4.33	4.22	9.78	5.89	8.00	9.22	6.33
	Rank	**1**	2	3	5	3	10	6	8	9	7
	+/ -/ =	~	7/0/2	9/0/0	9/0/0	8/0/1	9/0/0	9/0/0	9/0/0	9/0/0	9/0/0
20	Avg	**1.00**	2.22	4.22	3.89	4.44	9.67	5.78	8.00	9.33	6.44
	Rank	**1**	2	4	3	5	10	6	8	9	7
	+/ -/ =	~	9/0/0	9/0/0	9/0/0	9/0/0	9/0/0	9/0/0	9/0/0	9/0/0	9/0/0

Table A.4. Kapur's entropy obtained by EACOR and similar algorithms on the melanoma images

Images	Levels	EACOR	ACOR	CS	GWO	HHO	SCA	ACWOA	IGWO	SCADE	m_SCA
Ⅰ	4	**3.7741E+01**	3.7699E+01	3.7612E+01	3.7601E+01	3.7695E+01	3.6078E+01	3.7634E+01	3.7619E+01	3.6572E+01	3.7588E+01
	8	5.9748E+01	**5.9750E+01**	5.9056E+01	5.9123E+01	5.9090E+01	5.5006E+01	5.8733E+01	5.8569E+01	5.5719E+01	5.9240E+01
	12	**7.7574E+01**	7.7464E+01	7.5115E+01	7.6117E+01	7.5513E+01	7.0013E+01	7.4808E+01	7.4075E+01	6.9180E+01	7.5996E+01
	16	**9.2001E+01**	9.1803E+01	9.1017E+01	8.9598E+01	9.1006E+01	8.1265E+01	8.7843E+01	8.7815E+01	7.8160E+01	8.8968E+01
	20	**1.0445E+02**	1.0315E+02	1.0070E+02	1.0283E+02	9.9690E+01	8.9542E+01	1.0107E+02	9.8571E+01	9.0069E+01	1.0029E+02
Ⅱ	4	**3.8211E+01**	3.7442E+01	3.8123E+01	3.8048E+01	3.8198E+01	3.6862E+01	3.7905E+01	3.8028E+01	3.6522E+01	3.8023E+01
	8	6.0123E+01	**6.0127E+01**	5.9626E+01	5.9525E+01	5.9740E+01	5.4494E+01	5.9330E+01	5.8987E+01	5.5029E+01	5.9177E+01
	12	**7.7789E+01**	7.7592E+01	7.6157E+01	7.5787E+01	7.6702E+01	6.9151E+01	7.5042E+01	7.4381E+01	7.0323E+01	7.4928E+01
	16	**9.2639E+01**	9.2036E+01	8.9673E+01	9.0472E+01	9.1095E+01	7.9851E+01	8.9325E+01	8.6910E+01	8.0373E+01	9.0025E+01
	20	**1.0507E+02**	1.0356E+02	1.0090E+02	1.0137E+02	1.0116E+02	8.8745E+01	1.0068E+02	9.6693E+01	9.0133E+01	1.0179E+02
Ⅲ	4	3.9645E+01	**3.9645E+01**	3.9503E+01	3.9564E+01	3.9632E+01	3.9154E+01	3.9482E+01	3.9565E+01	3.7317E+01	3.9236E+01
	8	6.1509E+01	**6.1512E+01**	6.0928E+01	6.0808E+01	6.1374E+01	5.5435E+01	6.0166E+01	6.0599E+01	5.5638E+01	6.0619E+01
	12	**7.8856E+01**	7.8827E+01	7.7526E+01	7.7534E+01	7.7541E+01	6.9370E+01	7.6615E+01	7.5859E+01	7.1352E+01	7.6127E+01
	16	**9.3323E+01**	9.3233E+01	9.0792E+01	9.1123E+01	9.0742E+01	8.1128E+01	9.0489E+01	8.8070E+01	8.5077E+01	9.0442E+01
	20	**1.0543E+02**	1.0467E+02	1.0345E+02	1.0315E+02	1.0216E+02	8.9658E+01	1.0201E+02	9.8554E+01	9.2586E+01	1.0088E+02
Ⅳ	4	3.7588E+01	**3.7589E+01**	3.7498E+01	3.7452E+01	3.7509E+01	3.6435E+01	3.7072E+01	3.7551E+01	3.5777E+01	3.7382E+01

	8	**5.8583E+01**	5.8569E+01	5.7886E+01	5.8167E+01	5.8301E+01	5.4358E+01	5.7317E+01	5.7539E+01	5.2706E+01	5.7818E+01
	12	7.5348E+01	**7.5380E+01**	7.3609E+01	7.4062E+01	7.4288E+01	6.7223E+01	7.3517E+01	7.2660E+01	6.7886E+01	7.3619E+01
	16	**8.9699E+01**	8.9498E+01	8.5891E+01	8.7036E+01	8.6916E+01	7.6644E+01	8.5789E+01	8.5045E+01	7.8367E+01	8.7632E+01
	20	**1.0180E+02**	1.0094E+02	9.7821E+01	9.8109E+01	9.7495E+01	8.8570E+01	9.6748E+01	9.4638E+01	8.5935E+01	9.6671E+01
Ⅴ	4	**3.5908E+01**	3.5908E+01	3.5879E+01	3.5907E+01	3.5902E+01	3.5309E+01	3.5877E+01	3.5899E+01	3.5633E+01	3.5897E+01
	8	5.6630E+01	**5.6634E+01**	5.6401E+01	5.6435E+01	5.6575E+01	5.3664E+01	5.6354E+01	5.6197E+01	5.3544E+01	5.6379E+01
	12	**7.3981E+01**	7.3878E+01	7.2923E+01	7.2896E+01	7.3614E+01	6.6932E+01	7.2262E+01	7.1676E+01	6.5509E+01	7.2635E+01
	16	8.8365E+01	**8.8542E+01**	8.6887E+01	8.7203E+01	8.7084E+01	7.7696E+01	8.5461E+01	8.3746E+01	7.7646E+01	8.6724E+01
	20	**1.0063E+02**	1.0008E+02	9.8043E+01	9.9075E+01	9.8616E+01	8.7862E+01	9.8697E+01	9.6178E+01	8.5901E+01	9.8359E+01
Ⅵ	4	**3.8058E+01**	3.8042E+01	3.8007E+01	3.7985E+01	3.7951E+01	3.6872E+01	3.7899E+01	3.8004E+01	3.6686E+01	3.7957E+01
	8	**5.9980E+01**	5.9965E+01	5.9667E+01	5.9374E+01	5.9680E+01	5.4997E+01	5.9502E+01	5.8857E+01	5.5746E+01	5.9312E+01
	12	**7.7709E+01**	7.7590E+01	7.5868E+01	7.6052E+01	7.5801E+01	6.9452E+01	7.5780E+01	7.5326E+01	6.8130E+01	7.5379E+01
	16	**9.2364E+01**	9.2148E+01	8.9854E+01	9.1265E+01	9.0275E+01	7.9615E+01	8.7705E+01	8.6812E+01	8.1534E+01	8.9355E+01
	20	**1.0450E+02**	1.0379E+02	1.0088E+02	1.0189E+02	1.0231E+02	9.1282E+01	1.0098E+02	9.7516E+01	9.3585E+01	1.0032E+02
Ⅶ	4	3.5892E+01	3.5892E+01	3.5796E+01	3.5881E+01	3.5796E+01	3.5461E+01	3.5756E+01	**3.5906E+01**	3.5010E+01	3.5873E+01
	8	**5.7131E+01**	5.7064E+01	5.6071E+01	5.6764E+01	5.6670E+01	5.2615E+01	5.6045E+01	5.6627E+01	5.1403E+01	5.6603E+01
	12	**7.4043E+01**	7.3593E+01	7.2315E+01	7.2910E+01	7.2949E+01	6.6372E+01	7.2239E+01	7.1162E+01	6.5936E+01	7.2602E+01
	16	**8.7833E+01**	8.7150E+01	8.5015E+01	8.5805E+01	8.6502E+01	7.7104E+01	8.5999E+01	8.3335E+01	7.7809E+01	8.5902E+01
	20	**9.9675E+01**	9.8189E+01	9.6599E+01	9.7397E+01	9.7582E+01	8.4201E+01	9.7507E+01	9.3215E+01	8.5848E+01	9.6479E+01
Ⅷ	4	**3.6429E+01**	**3.6429E+01**	3.6410E+01	3.6425E+01	3.6425E+01	3.5711E+01	3.6413E+01	3.6423E+01	3.5738E+01	3.6427E+01
	8	5.7067E+01	**5.7088E+01**	5.6793E+01	5.7031E+01	5.7040E+01	5.3521E+01	5.6587E+01	5.6662E+01	5.3703E+01	5.6944E+01
	12	7.3769E+01	**7.3955E+01**	7.2860E+01	7.3455E+01	7.3501E+01	6.7130E+01	7.2680E+01	7.1964E+01	6.6224E+01	7.2260E+01
	16	**8.8640E+01**	8.8397E+01	8.7103E+01	8.7238E+01	8.7223E+01	7.6125E+01	8.5858E+01	8.4935E+01	7.7659E+01	8.6823E+01
	20	**1.0107E+02**	1.0012E+02	9.7285E+01	9.9138E+01	9.8625E+01	9.0902E+01	9.9882E+01	9.3802E+01	8.8859E+01	9.6926E+01
Ⅸ	4	**3.8294E+01**	**3.8294E+01**	3.8139E+01	3.8191E+01	3.8161E+01	3.6522E+01	3.8175E+01	3.7963E+01	3.6280E+01	3.8063E+01
	8	6.0035E+01	**6.0047E+01**	5.9413E+01	5.9341E+01	5.9842E+01	5.6175E+01	5.9075E+01	5.8666E+01	5.4919E+01	5.9393E+01
	12	**7.7729E+01**	7.7710E+01	7.5965E+01	7.6653E+01	7.6154E+01	7.0770E+01	7.5328E+01	7.5109E+01	7.0757E+01	7.5636E+01
	16	**9.2381E+01**	9.2365E+01	8.9111E+01	9.0152E+01	8.9558E+01	8.0605E+01	8.9008E+01	8.8054E+01	7.9962E+01	8.9580E+01
	20	**1.0500E+02**	1.0438E+02	1.0218E+02	1.0218E+02	1.0180E+02	8.9475E+01	1.0071E+02	9.7057E+01	9.0659E+01	1.0059E+02

Appendix B

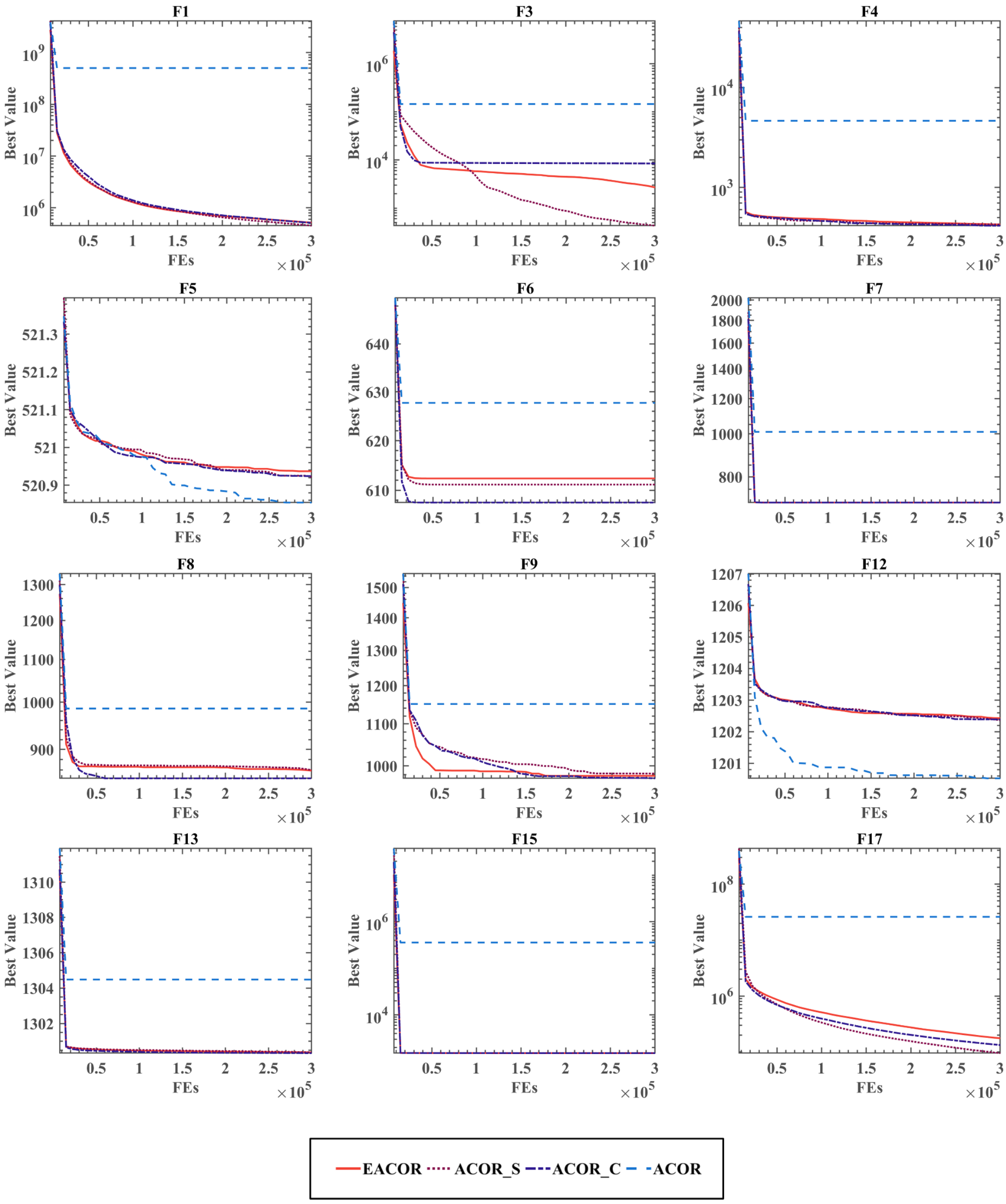

Figure B.1. The convergence curve of the EACOR strategy combination

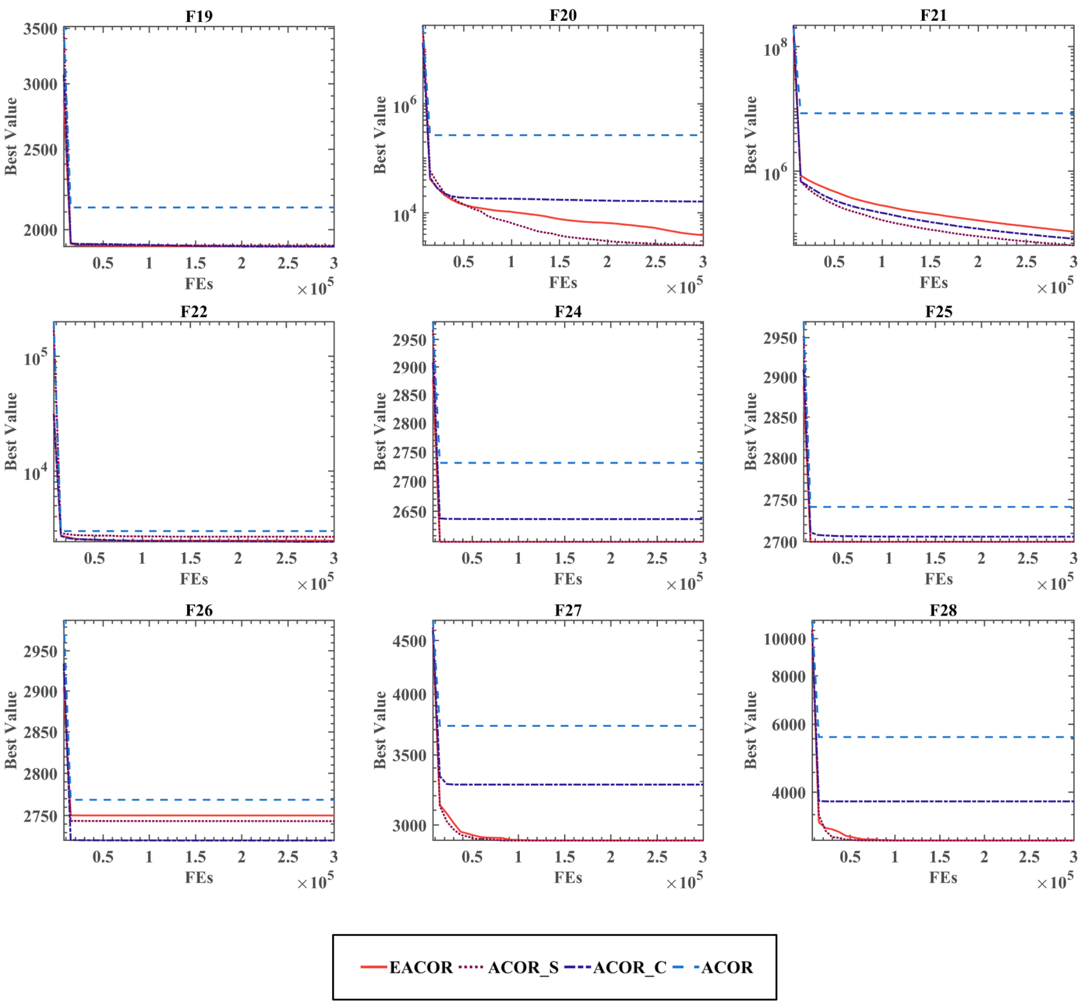

Figure B.2. The convergence curve of the EACOR strategy combination

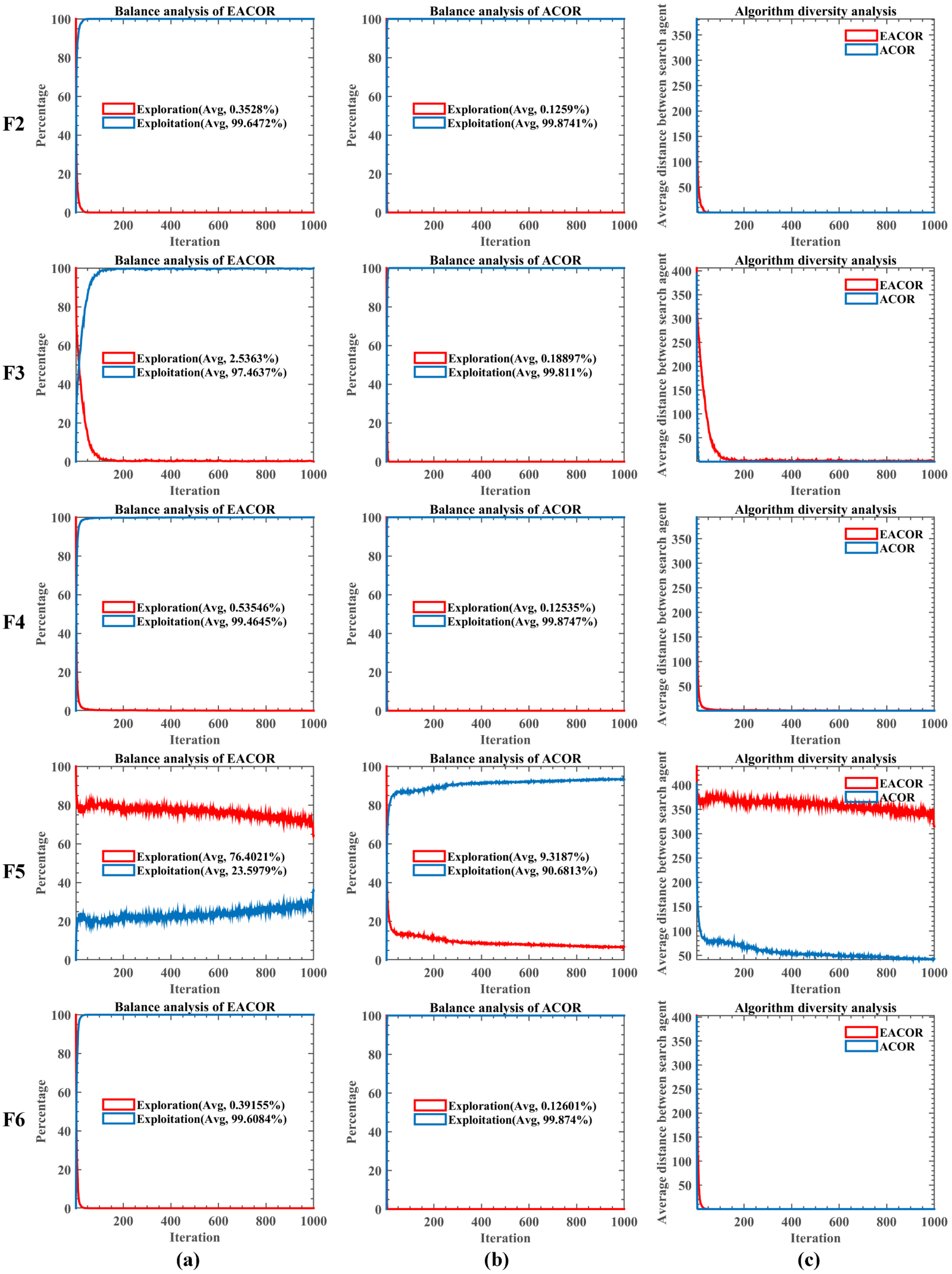

Figure B.3. (a) The balance tests of EACOR (b) The balance tests of ACOR (c) The diversity tests of EACOR and ACOR

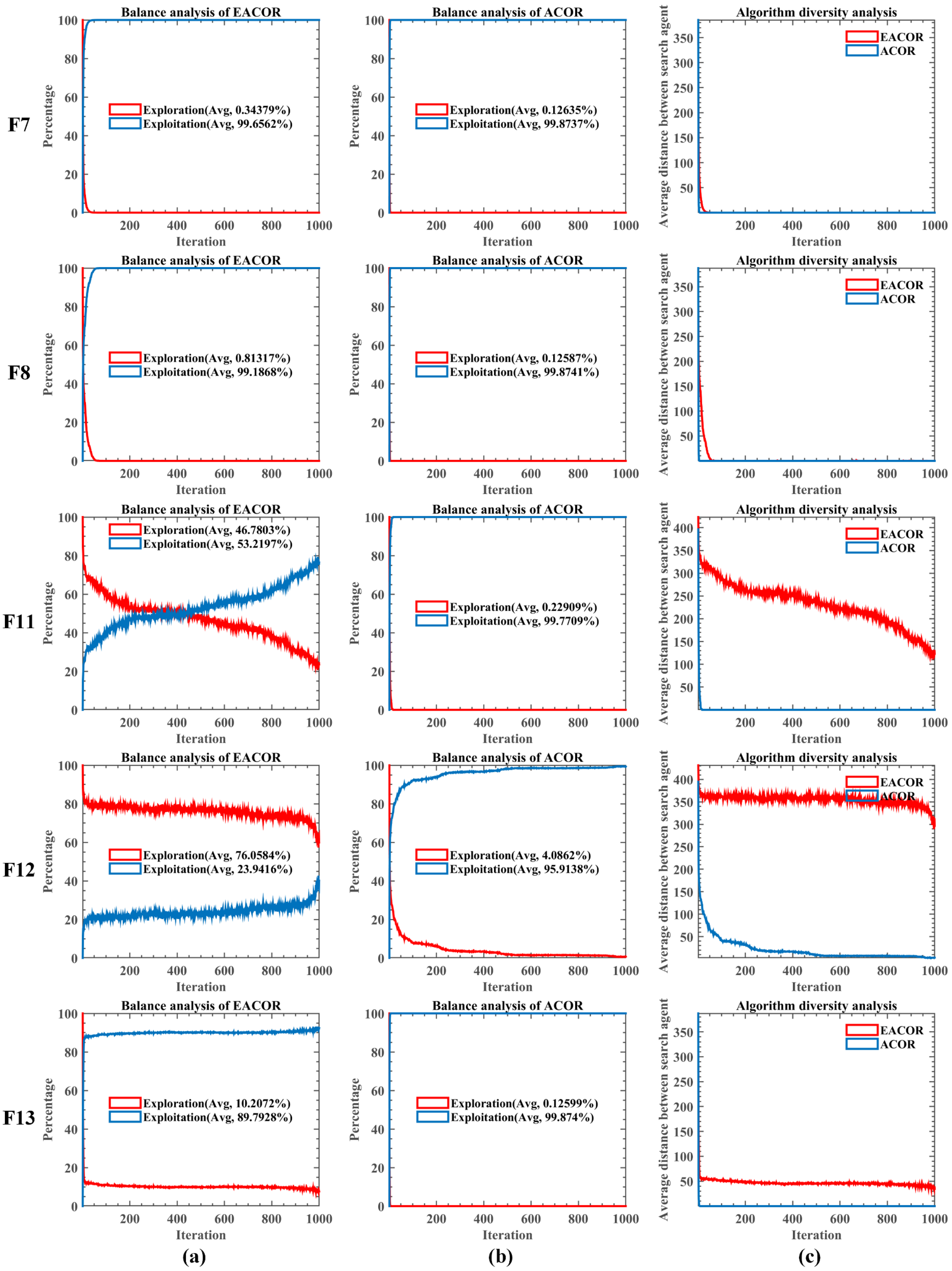

Figure B.4. (a) The balance tests of EACOR (b) The balance tests of ACOR (c) The diversity tests of EACOR and ACOR

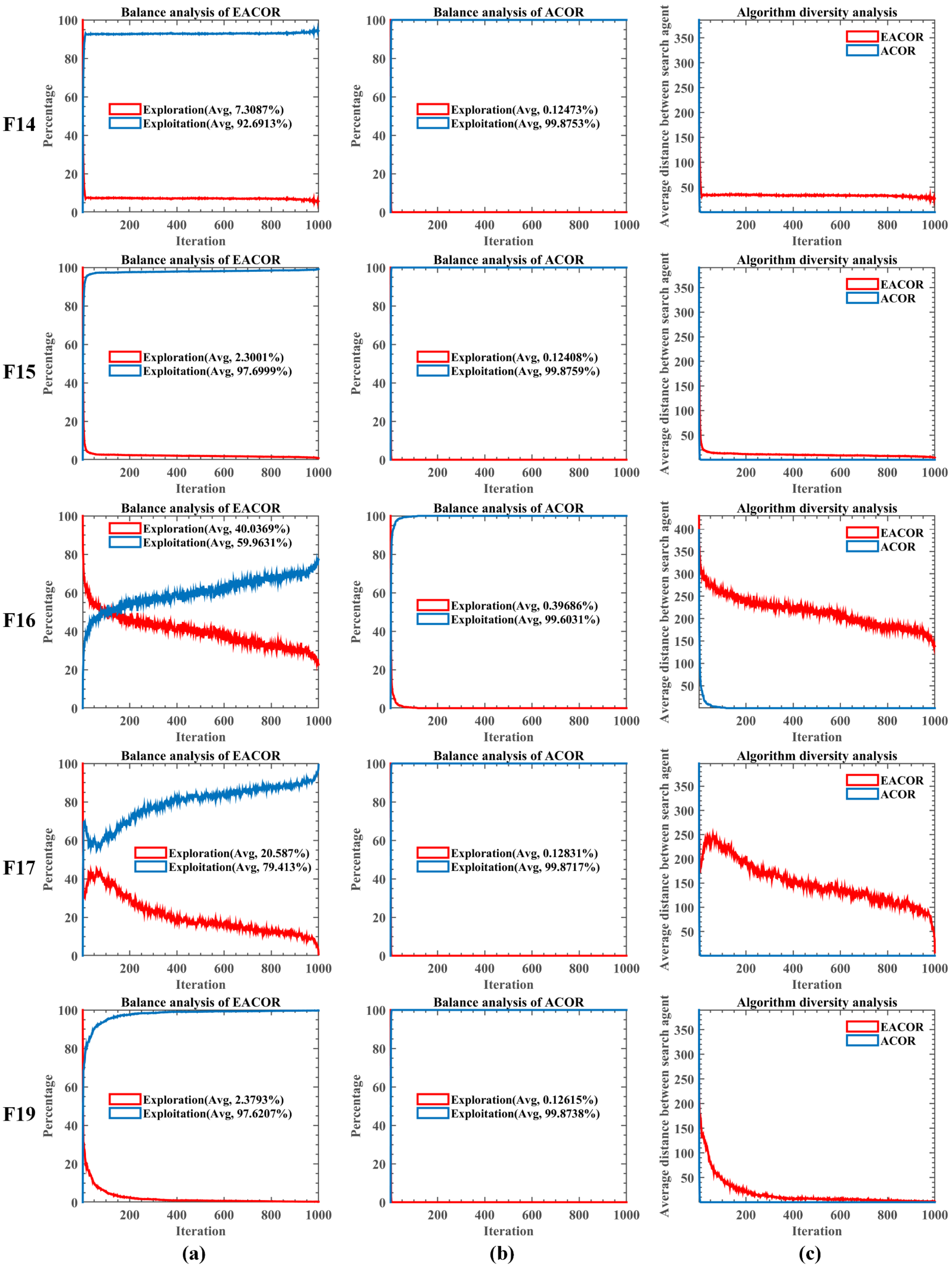

Figure B.5. (a) The balance tests of EACOR (b) The balance tests of ACOR (c) The diversity tests of EACOR and ACOR

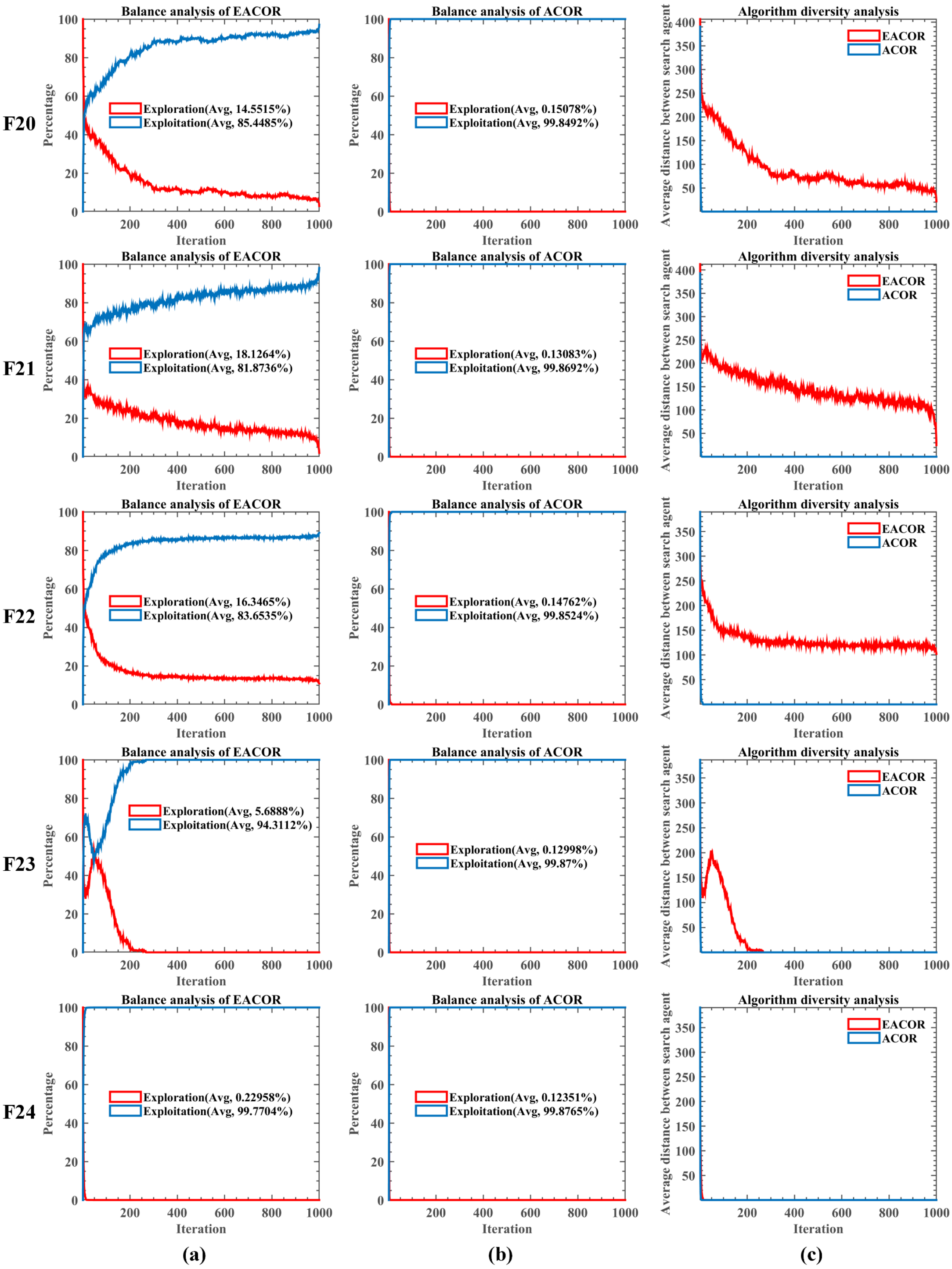

Figure B.6. (a) The balance tests of EACOR (b) The balance tests of ACOR (c) The diversity tests of EACOR and ACOR

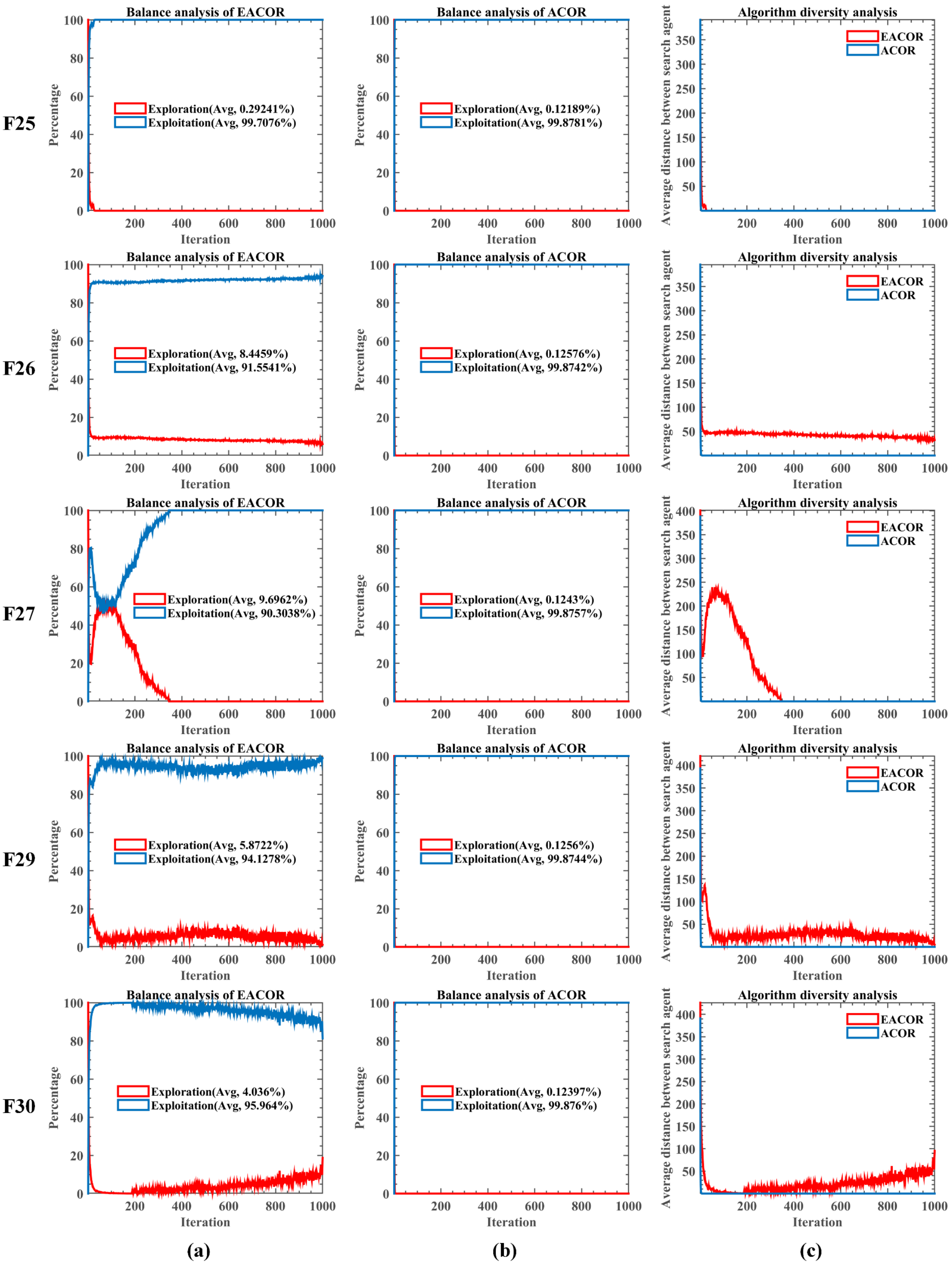

Figure B.7. (a) The balance tests of EACOR (b) The balance tests of ACOR (c) The diversity tests of EACOR and ACOR

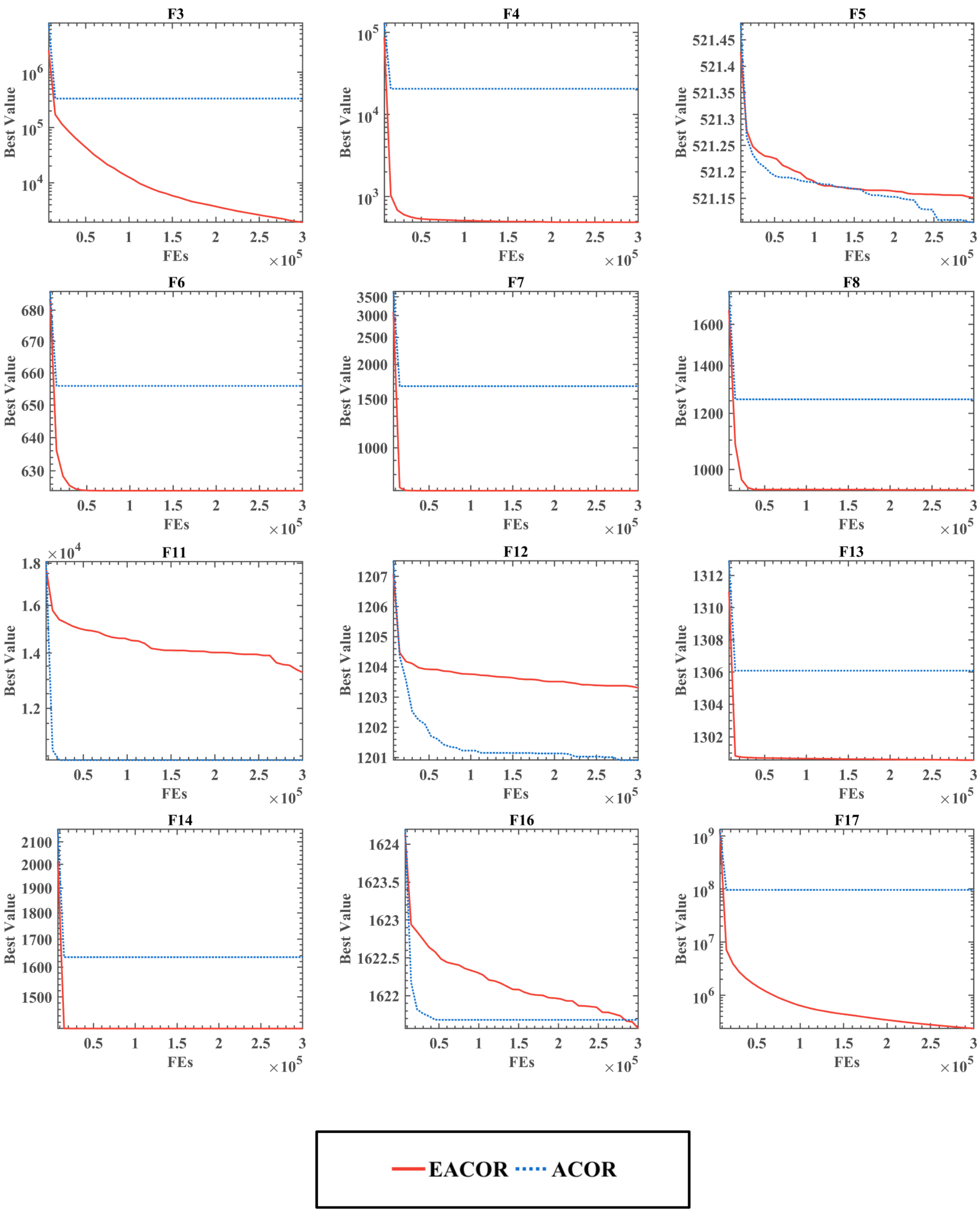

Figure B.8. Convergence curves of EACOR and ACOR at 50 dimensions

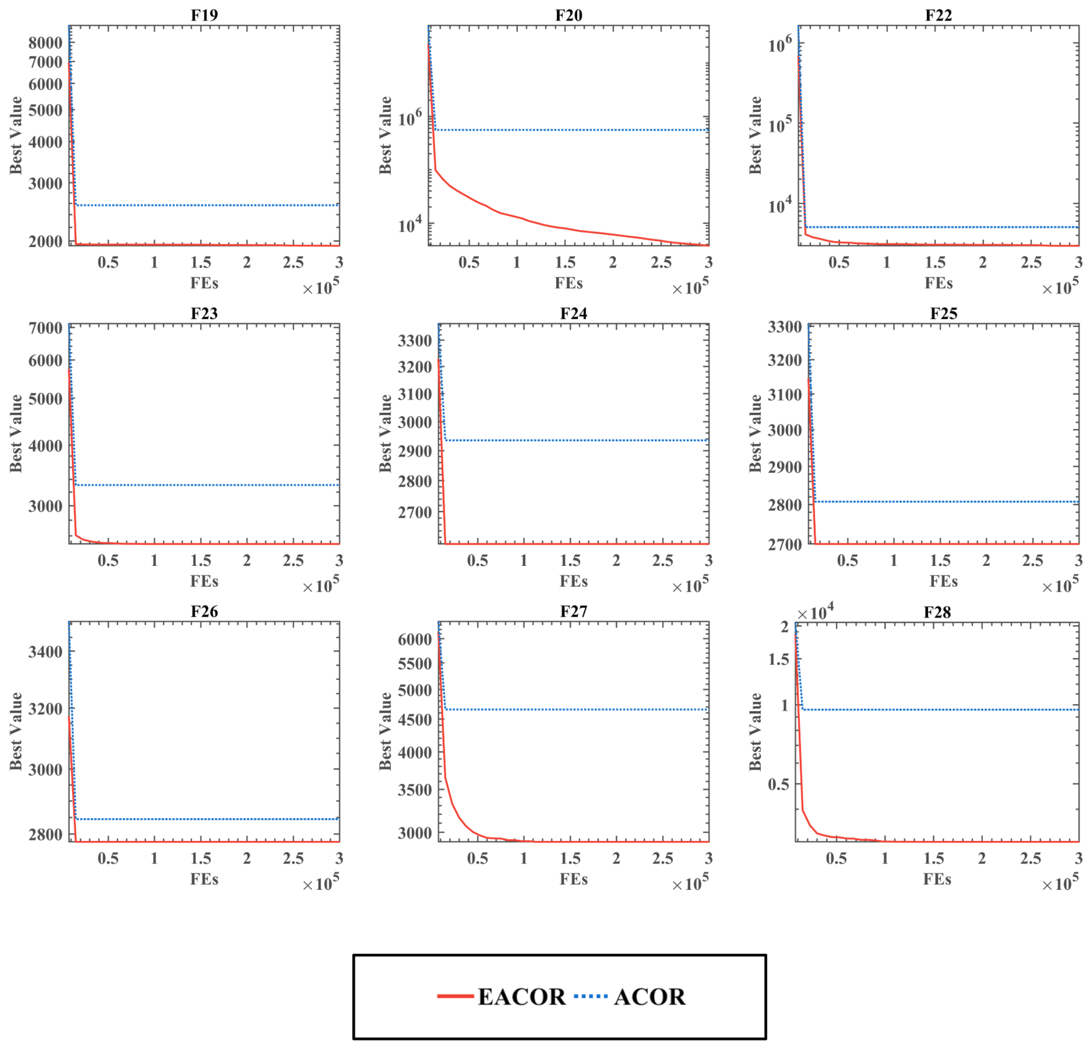

Figure B.9. Convergence curves of EACOR and ACOR at 50 dimensions

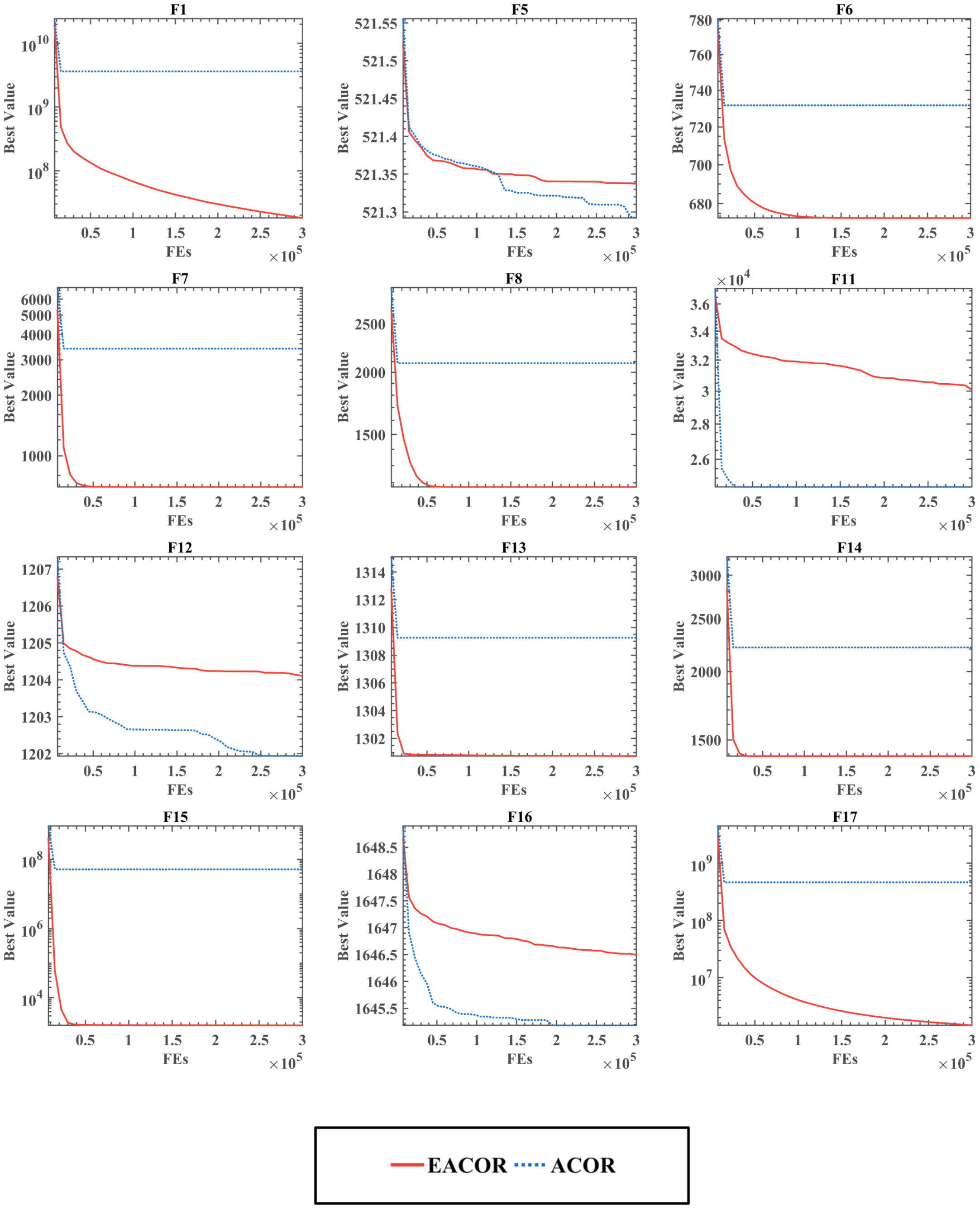

Figure B.10. Convergence curves of EACOR and ACOR at 100 dimensions

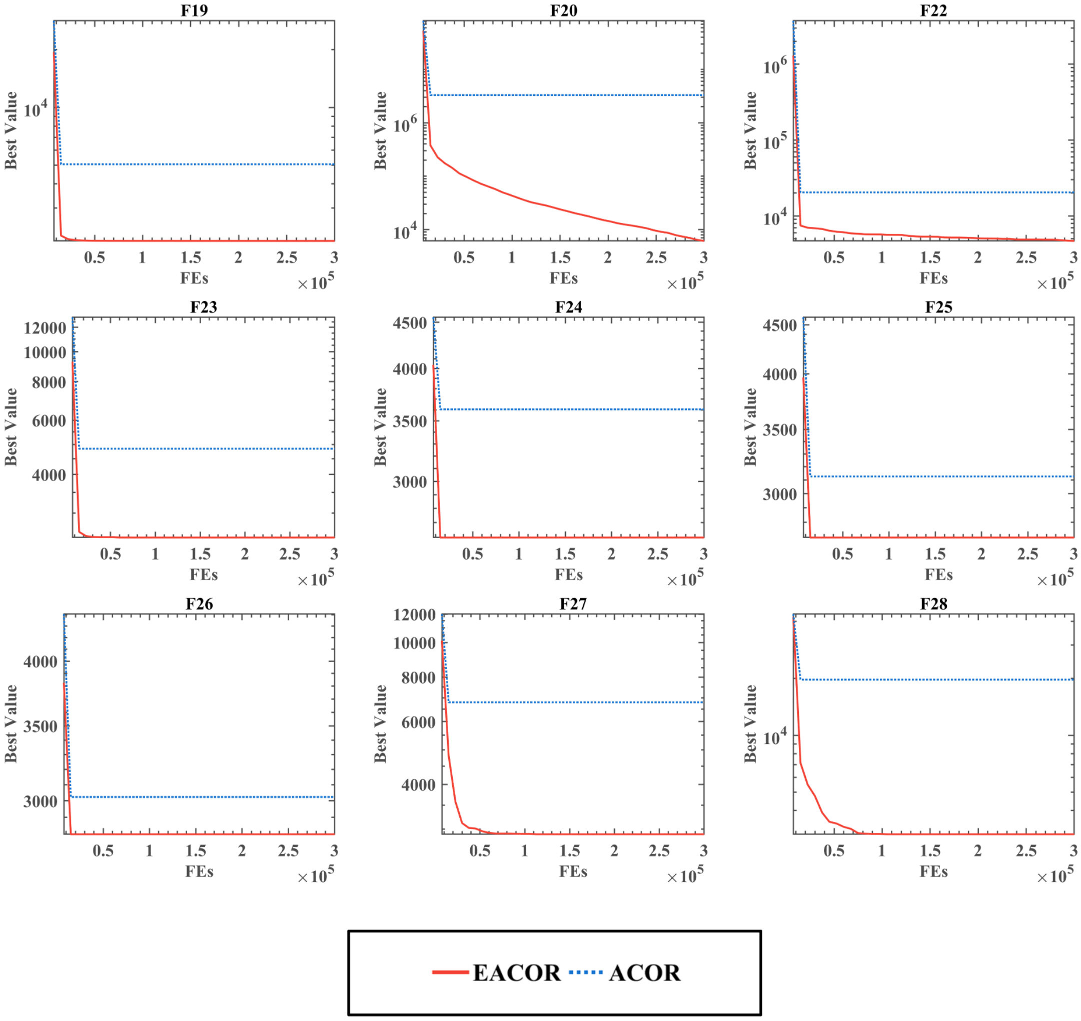

Figure B.11. Convergence curves of EACOR and ACOR at 100 dimensions

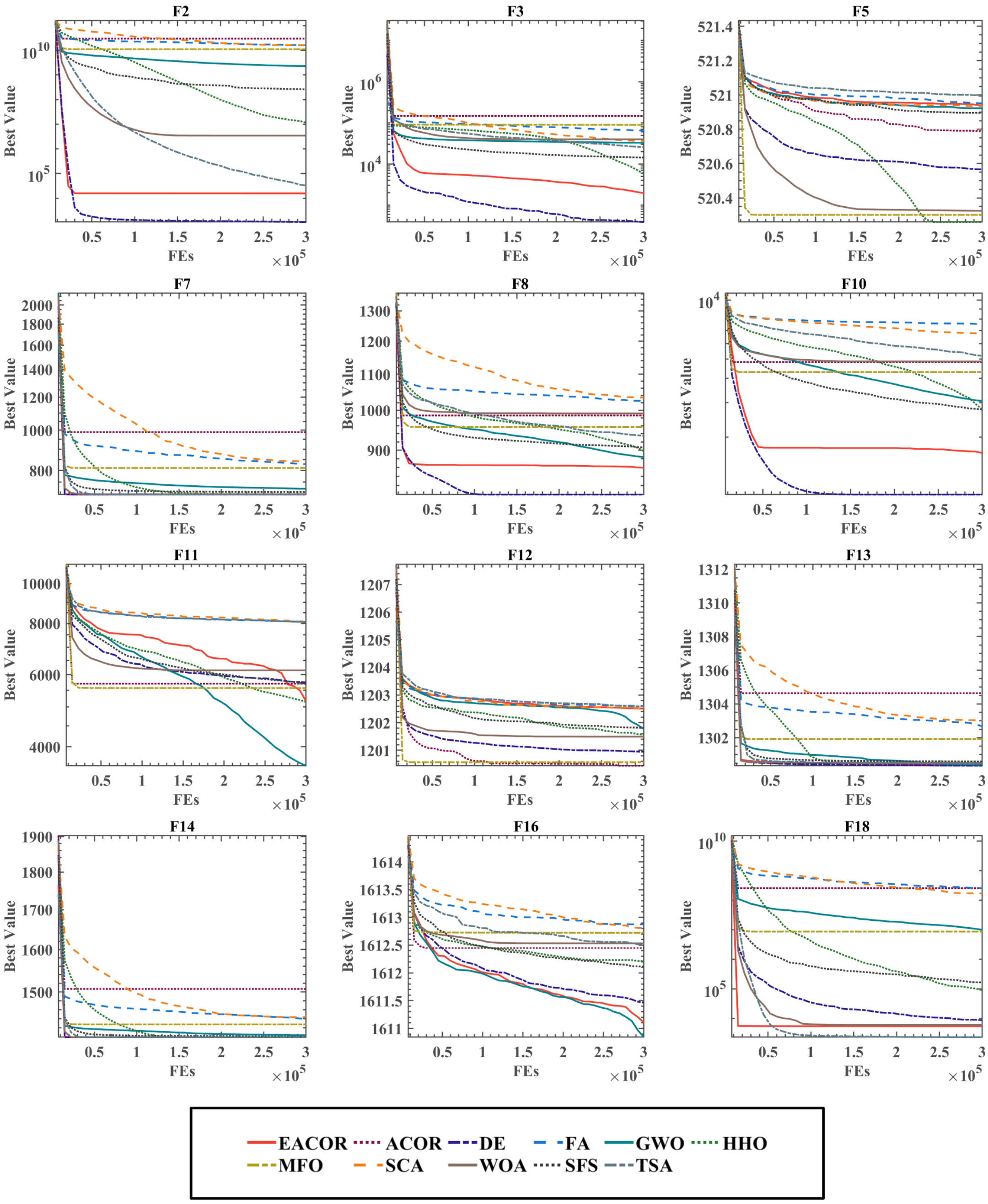

Figure B.12. Convergence curves of EACOR with ten original algorithms

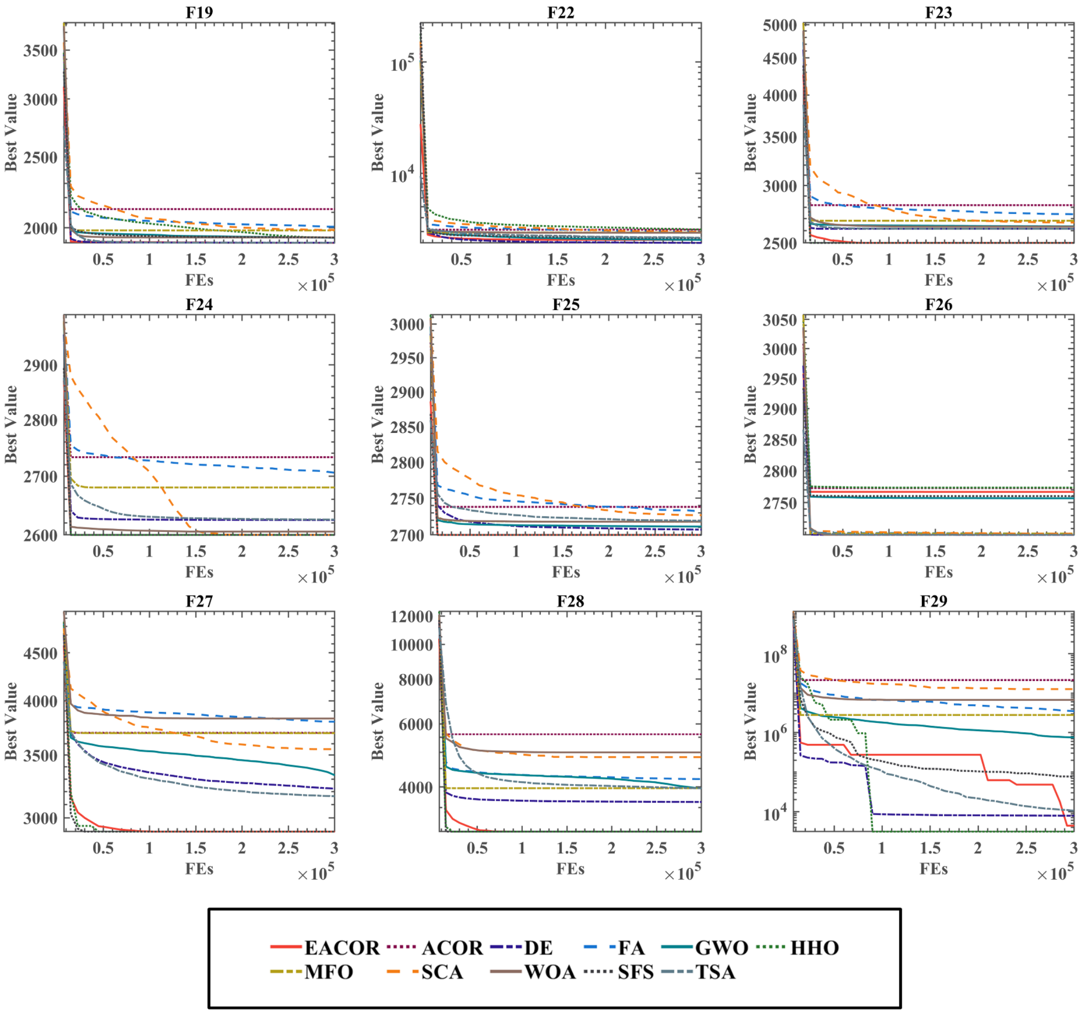

Figure B.13. Convergence curves of EACOR with ten original algorithms

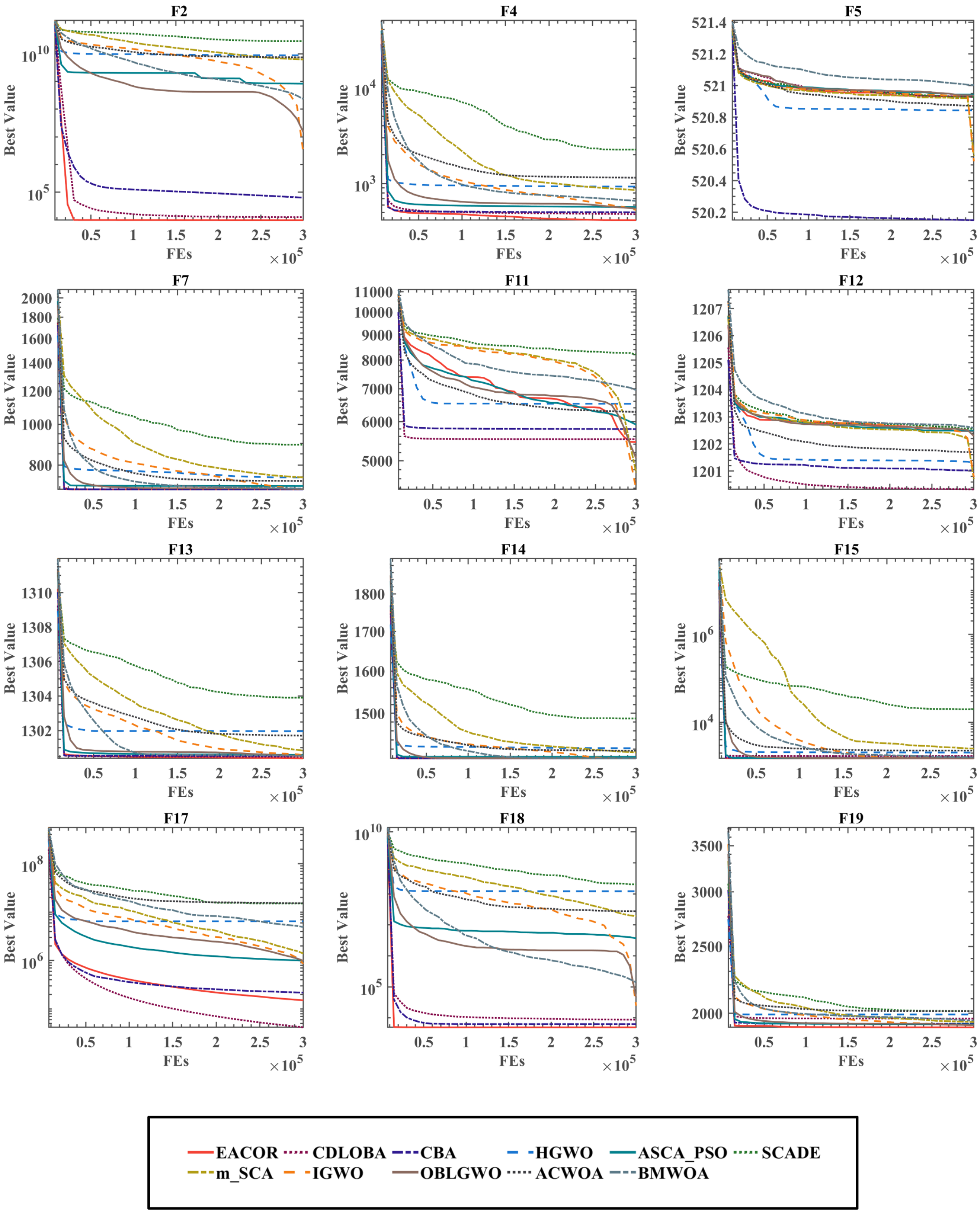

Figure B.14. Convergence curves of EACOR with ten peers

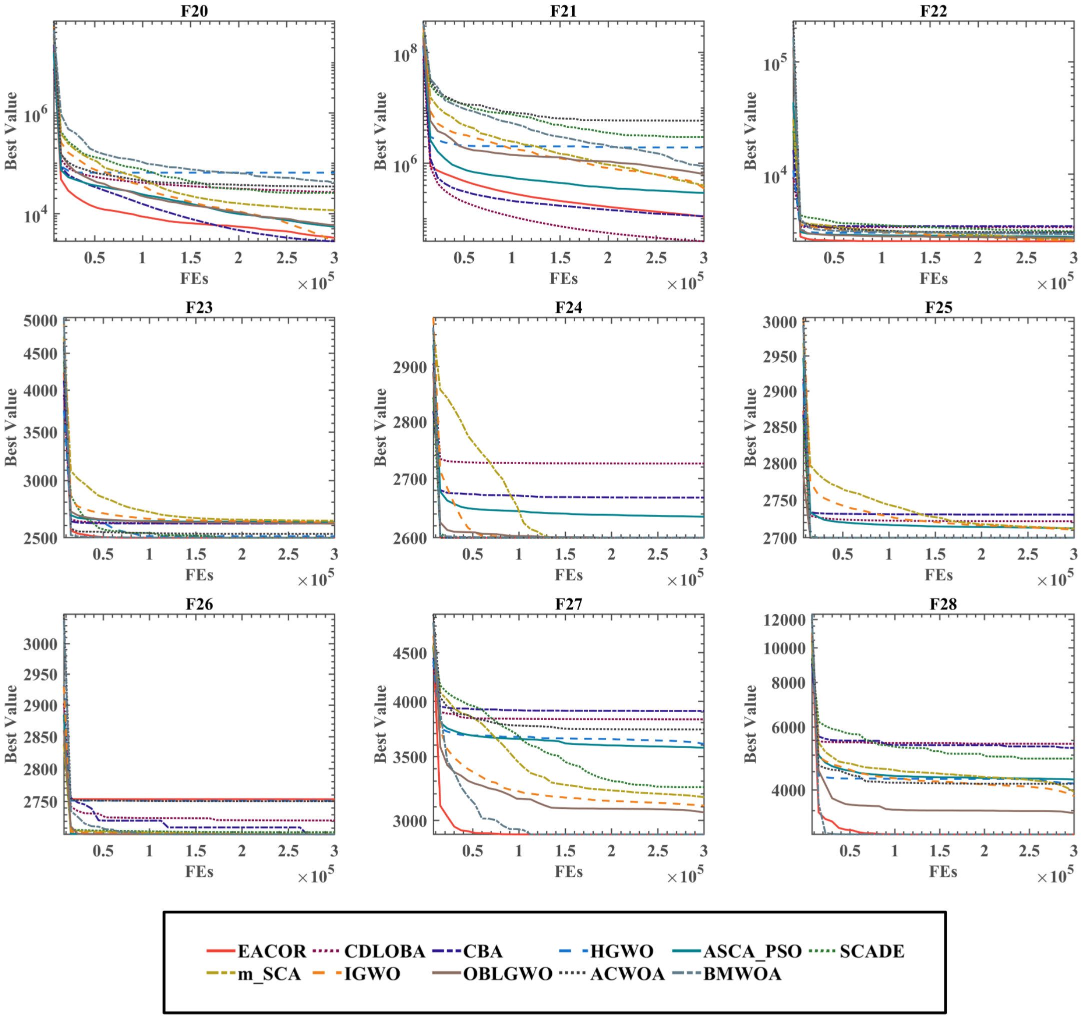

Figure B.15. Convergence curves of EACOR with ten peers

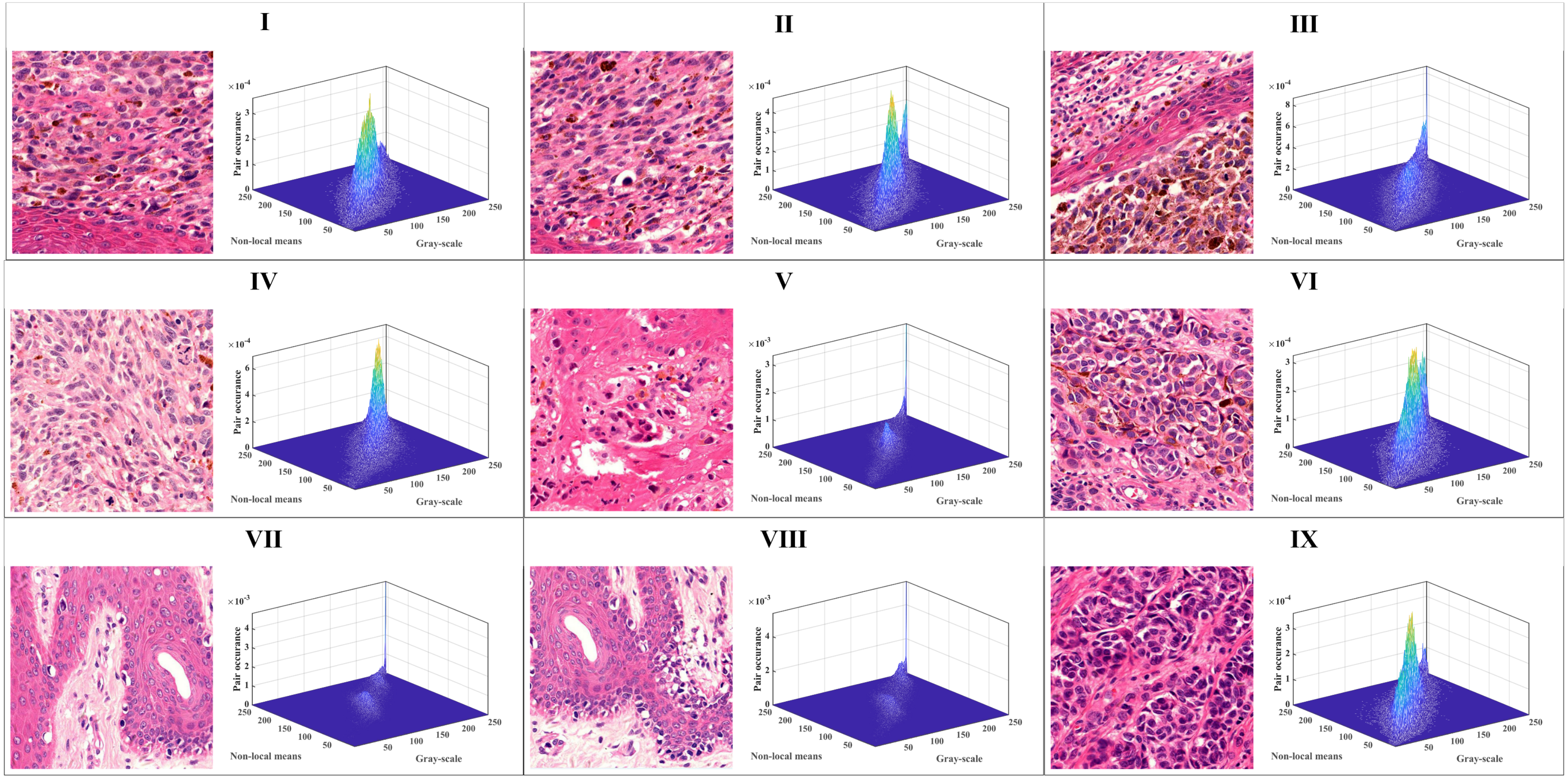

Figure B.16. Melanoma original image and non-local mean 2D histogram

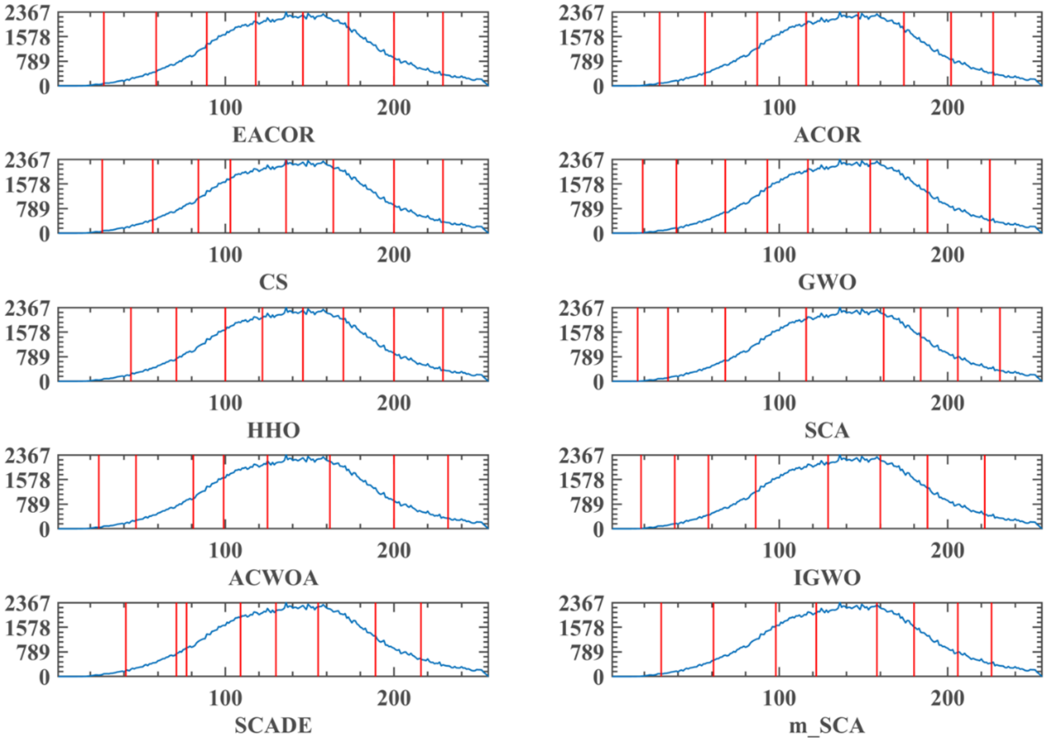

Figure B.17. Results of 8 level threshold segmentation of image Ⅰ by all algorithms

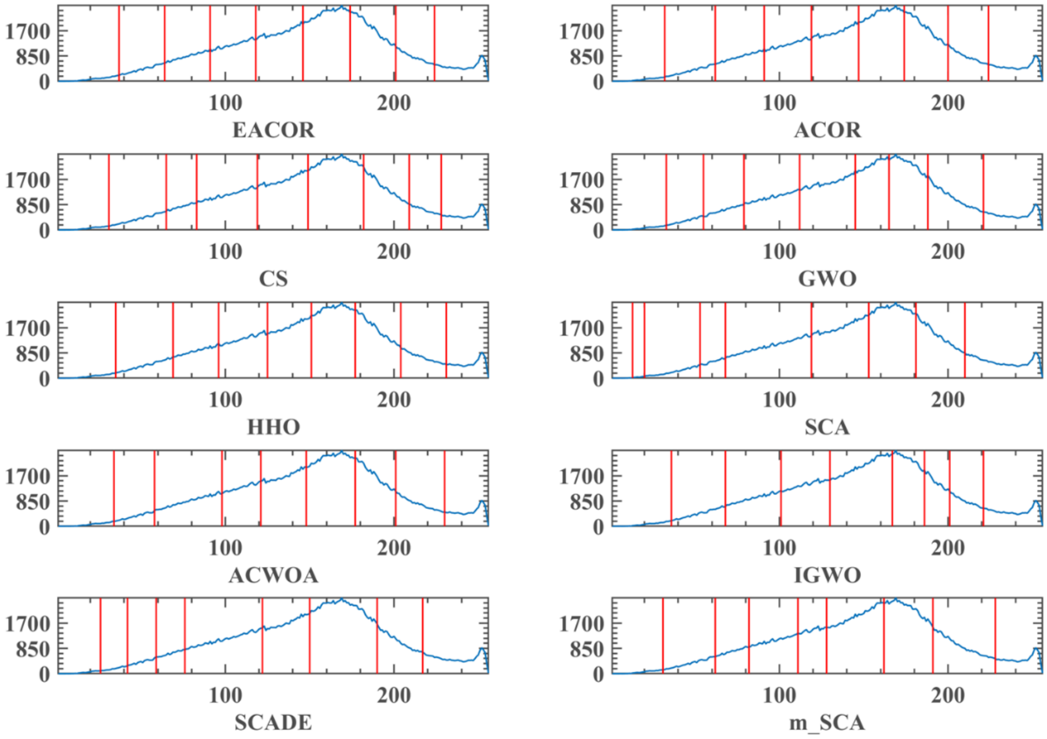

Figure B.18. Results of 8 level threshold segmentation of image Ⅱ by all algorithms

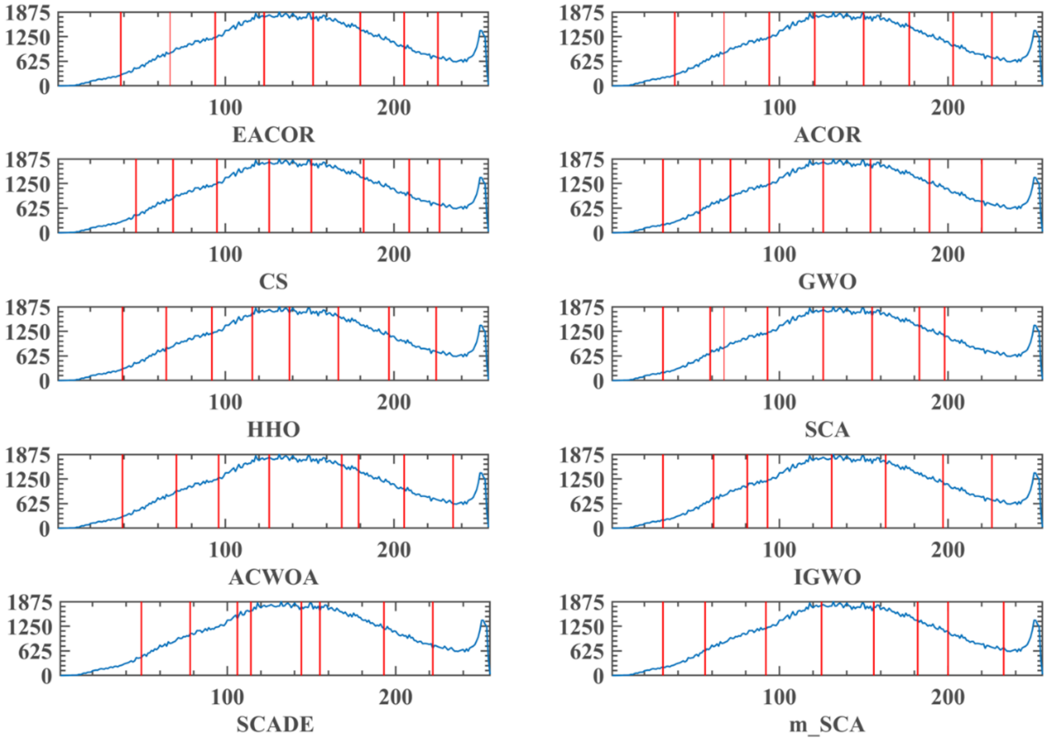

Figure B.19. Results of 8 level threshold segmentation of image Ⅲ by all algorithms

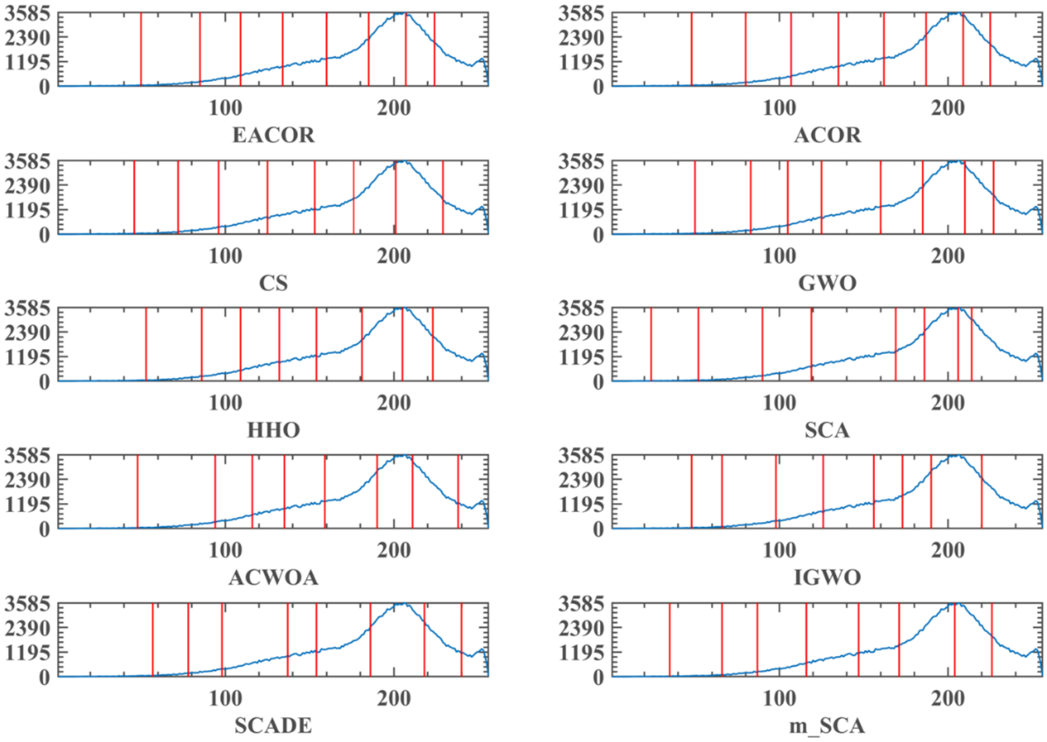

Figure B.20. Results of 8 level threshold segmentation of image Ⅳ by all algorithms

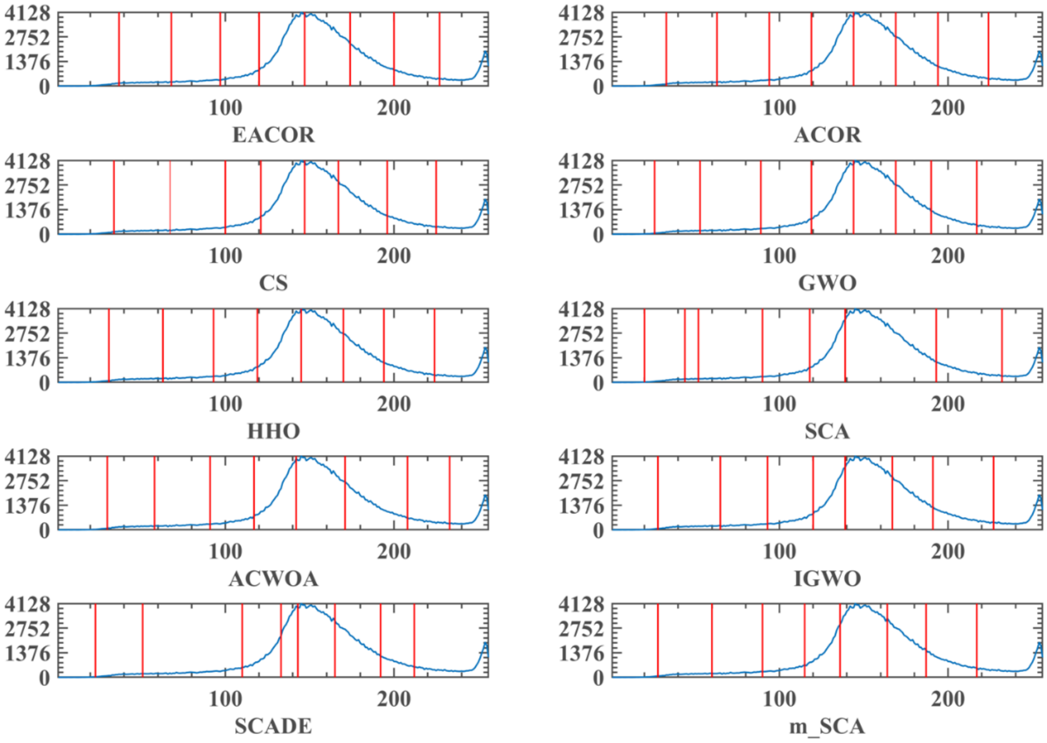

Figure B.21. Results of 8 level threshold segmentation of image Ⅴ by all algorithms

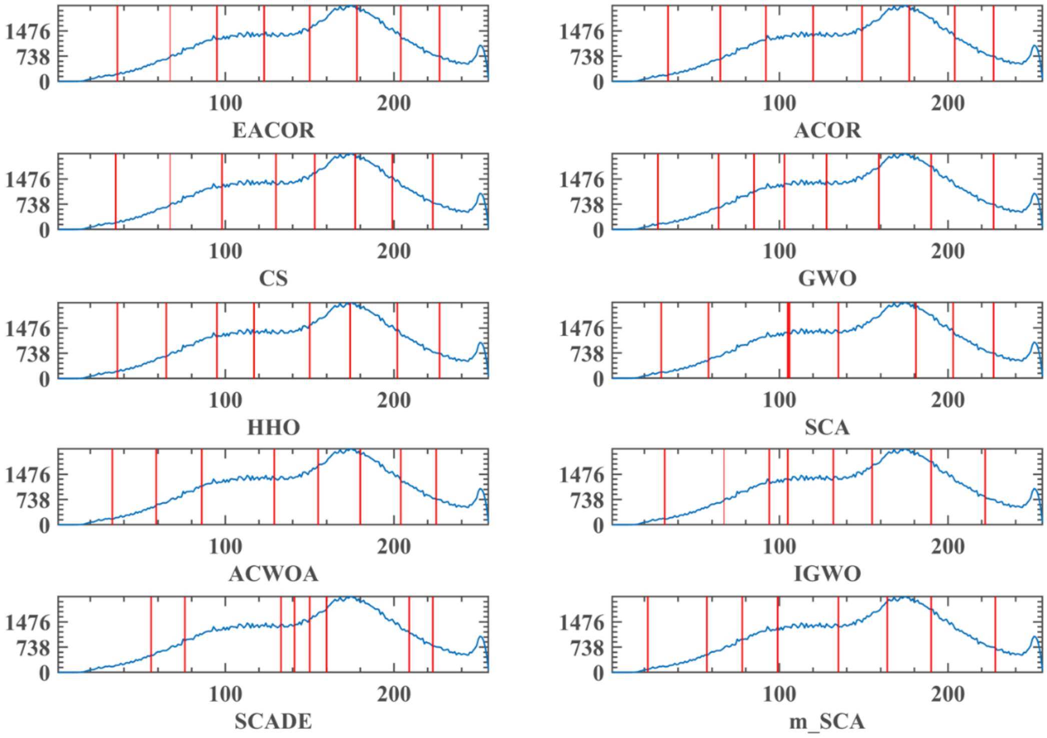

Figure B.22. Results of 8 level threshold segmentation of image Ⅵ by all algorithms

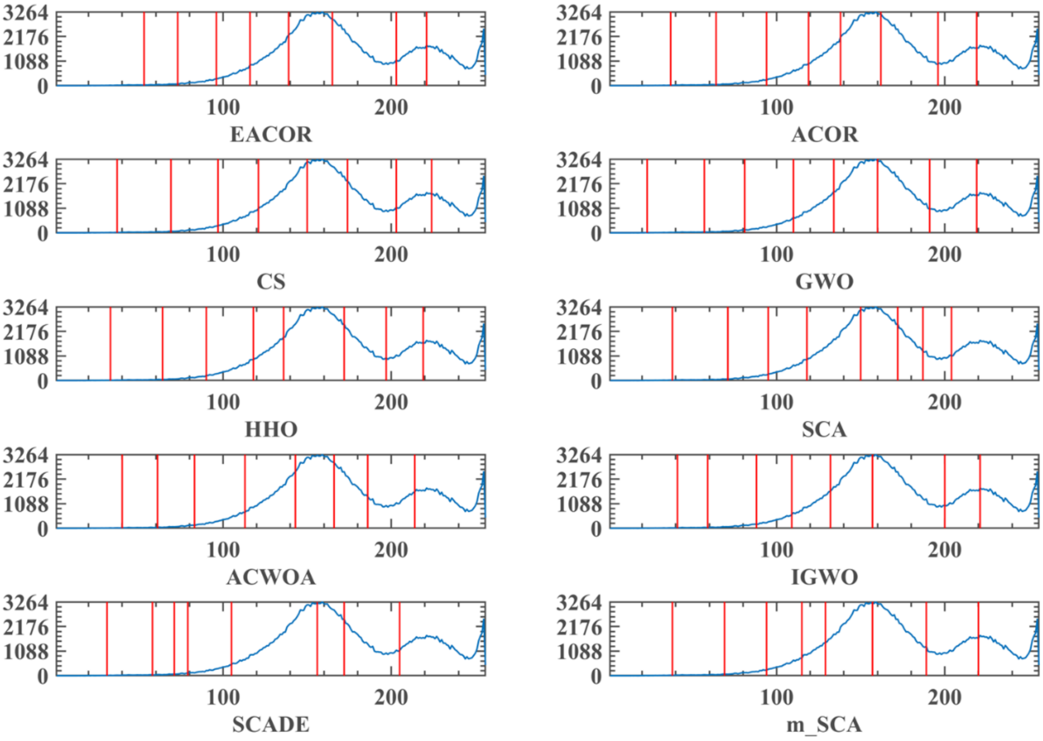

Figure B.23. Results of 8 level threshold segmentation of image Ⅶ by all algorithms

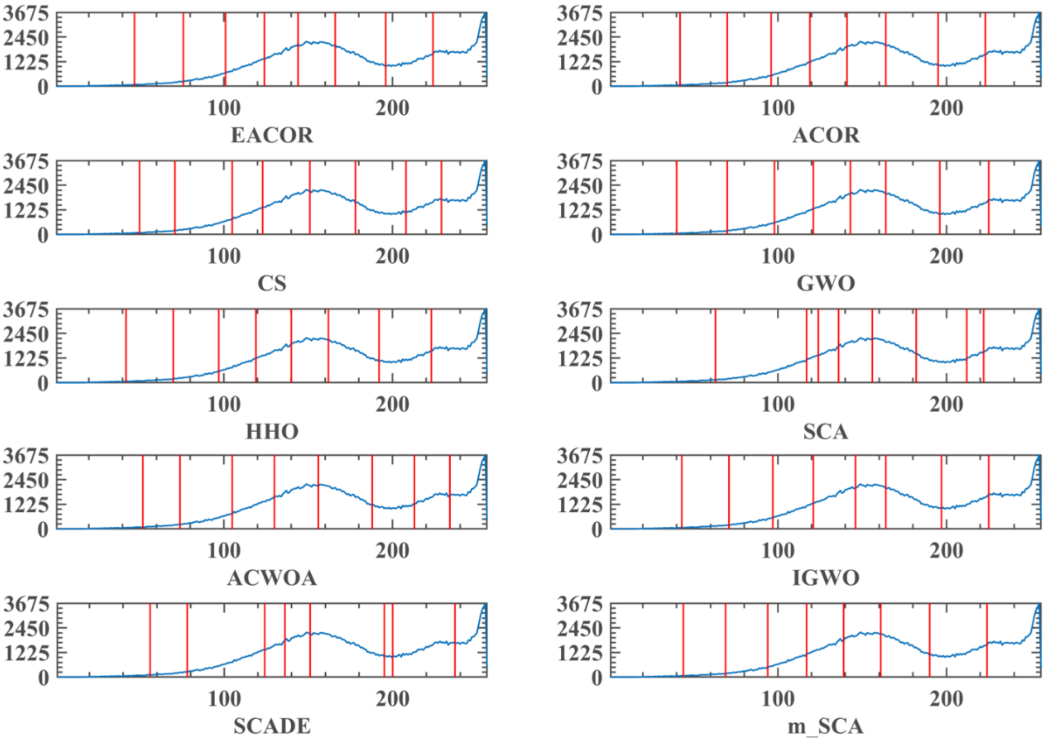

Figure B.24. Results of 8 level threshold segmentation of image Ⅷ by all algorithms

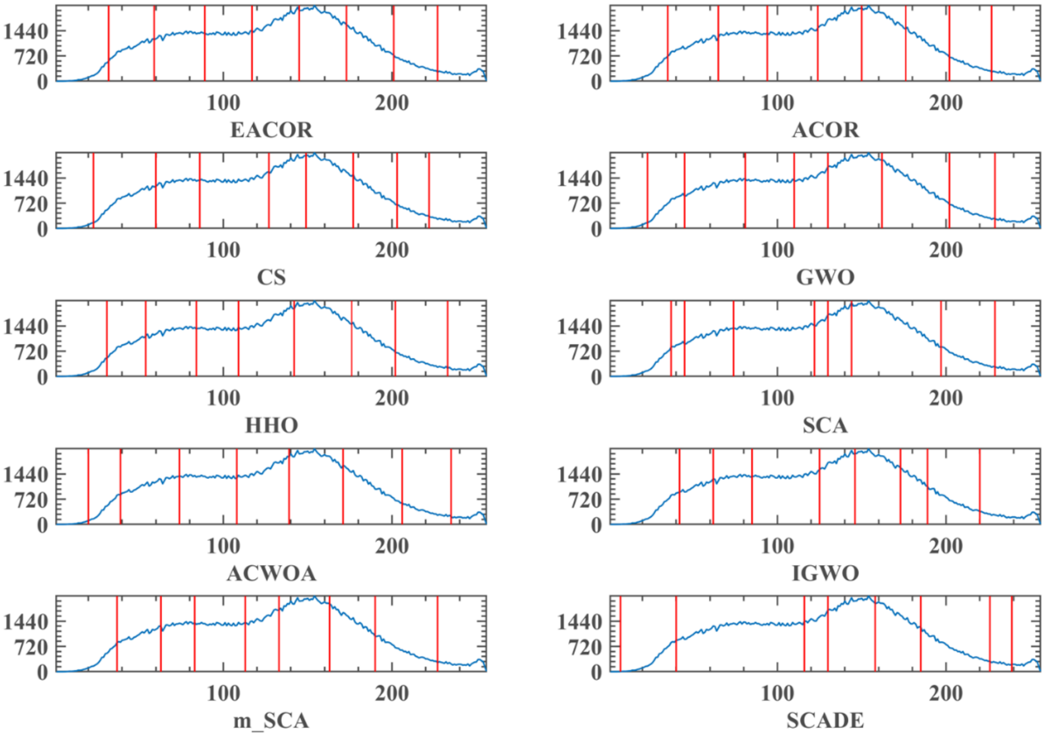

Figure B.25. Results of 8 level threshold segmentation of image Ⅸ by all algorithms
